# TransportTP: A two-phase classification approach for membrane transporter prediction and characterization

**DOI:** 10.1186/1471-2105-10-418

**Published:** 2009-12-14

**Authors:** Haiquan Li, Vagner A Benedito, Michael K Udvardi, Patrick Xuechun Zhao

**Affiliations:** 1 Plant Biology Division, The Samuel Roberts Noble Foundation, Inc, Ardmore, OK 73401, USA

## Abstract

**Background:**

Membrane transporters play crucial roles in living cells. Experimental characterization of transporters is costly and time-consuming. Current computational methods for transporter characterization still require extensive curation efforts, especially for eukaryotic organisms. We developed a novel genome-scale transporter prediction and characterization system called TransportTP that combined homology-based and machine learning methods in a two-phase classification approach. First, traditional homology methods were employed to predict novel transporters based on sequence similarity to known classified proteins in the Transporter Classification Database (TCDB). Second, machine learning methods were used to integrate a variety of features to refine the initial predictions. A set of rules based on transporter features was developed by machine learning using well-curated proteomes as guides.

**Results:**

In a cross-validation using the yeast proteome for training and the proteomes of ten other organisms for testing, TransportTP achieved an equivalent recall and precision of 81.8%, based on TransportDB, a manually annotated transporter database. In an independent test using the Arabidopsis proteome for training and four recently sequenced plant proteomes for testing, it achieved a recall of 74.6% and a precision of 73.4%, according to our manual curation.

**Conclusions:**

TransportTP is the most effective tool for eukaryotic transporter characterization up to date.

## Background

Membrane transporter proteins, or simply transporters, play crucial roles in living cells, such as importing essential nutrients and exporting toxic cellular metabolites, mediating signal transduction, and maintaining ionic and osmotic homeostasis.

A handful of in-vitro or in-vivo experimental methods have been developed and applied to study transporter mechanisms, such as the patch-clamp techniques for analyzing ion channels [1] and heterologous expression and mutant complementation approaches, which are often coupled with the use of isotopically-labeled substrates [2,3]. These methods are costly and time-consuming, both of which limit their applications in identifying transporters on a large scale. Therefore, computational methods are desired for selecting and sorting potential targets on a genome scale prior to laboratory experiments. Homology searches against experimentally-determined transporters is heretofore the most common approach in inferring novel transporters, as exemplified by BLAST searches [4] against the Transporter Classification Database (TCDB) [5,6]. Employing this approach, a putative transporter database named TransportDB was constructed for hundreds of completely sequenced genomes [7,8]. The wide adoption of TCDB for transporter annotation is due to its unique characteristics. It contains comprehensive information on experimentally-characterized transporters. These transporters are organized within a simple tree structure based on both function and homology, which contains over 550 transporter families. The transporter families possess a distinct functional-phylogenetic property, i.e. the members in a transporter family are not only homologous but also share similar transporter mechanisms. Other classification systems such as Pfam [9] and Gene Ontology [10] do not have this property. In the Pfam system, a specific domain may be contained within proteins of different functions. In the Gene Ontology system, a transporter may logically belong to multiple transporter terms or functions due to the Directed Acyclic Graphs (DAGs) adopted to represent relationships among protein functions.

Homology methods may reveal new putative transporters, but they can also generate many false positive assignments, due to systematic errors arising from homology inference, since nontrivial functional variations may be induced during gene duplication or domain shuffling [11,12]. For example, paralogs often exhibit distinct functions [13]. As a result, more complicated modeling of transporter families have been proposed, including profile based methods like HMMER [9] and PST [14], and machine learning methods like SVMProt [15]. Profile based methods rely heavily on regions of local conservation among family members. However, the level of conservation within many transporter families, such as potassium channels, may be very low [16,17], which limits the effectiveness of these methods. Machine learning methods can sidestep lack of conservation by utilizing quite distinct features such as physicochemical properties and overall composition of amino acids [15,18,19]. However, both methods, especially the machine learning ones, require many examples of a transporter family for effective modeling, which may be a limitation for many transporter families with few experimentally determined members.

Recently, some integrative methods have been reported that incorporate at least two data sources or methods. We proposed a nearest neighbor approach previously which integrated BLAST, Hidden Markov model (HMM) and topology analysis [20]. Another transporter annotation pipeline named TransAAP was launched along with TransportDB [8]. It searches TCDB [5], PFAM [9] and Gene Ontology [10], and utilizes a couple of empirical rules for decision. Though TransAAP currently works effectively for prokaryotic organisms, it is still weak in handling eukaryotic organisms. Therefore, existing computational tools for transporter prediction, including integrative methods, suffer from insufficient predictive coverage or low accuracy. Extensive curation efforts are still required for transporter annotation on a genome-wide scale.

**Figure 1:**
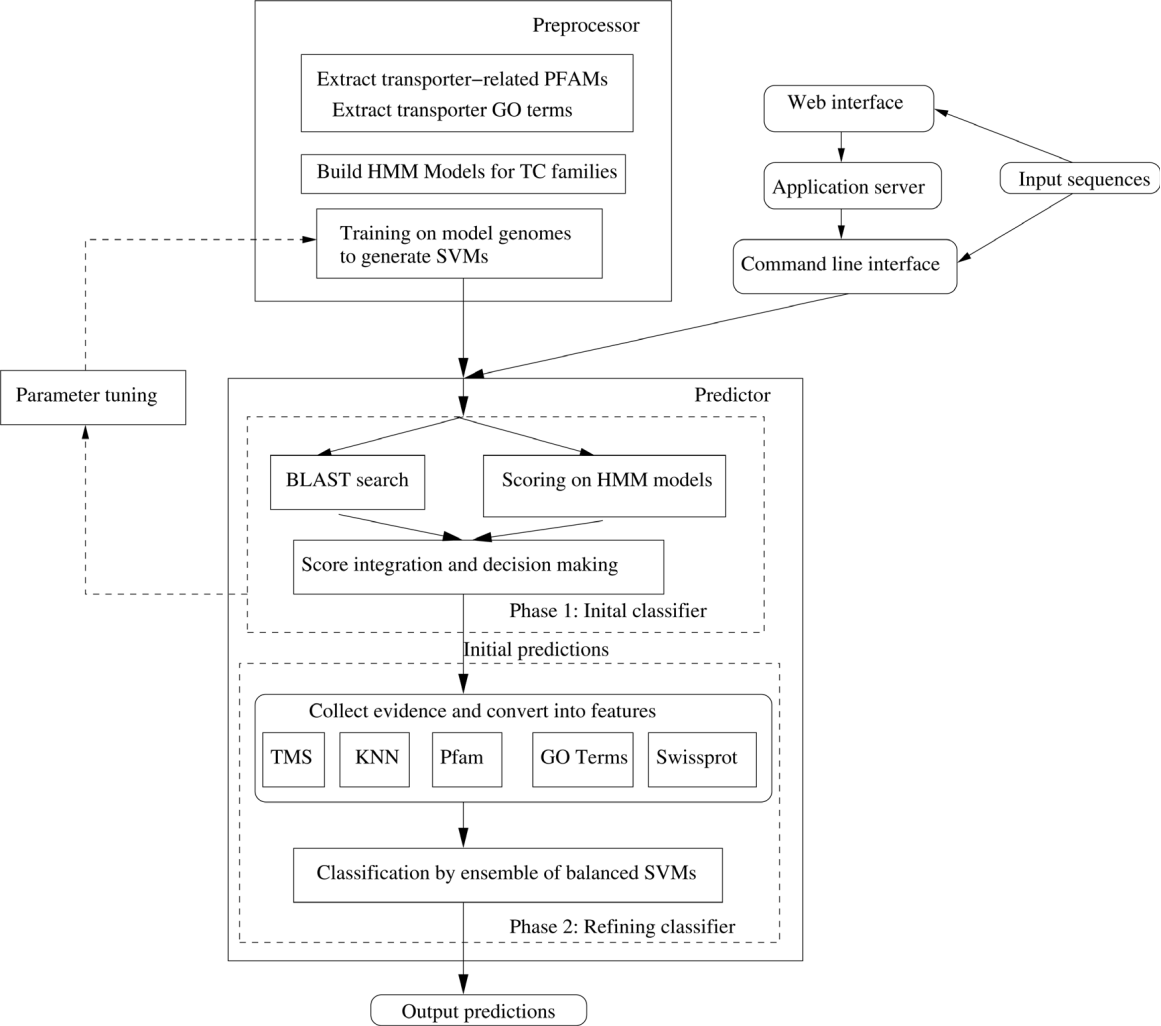
**The framework of the two-phase classifier TransportTP**.

Here, we present an automatic transporter prediction and characterization system called TransportTP which has significantly increased predictive performance than existing systems, including our previous work [20], requiring, therefore, much less manual curation. TransportTP utilizes a two-phase classification approach (Figure 1). Firstly, traditional homology-based approaches are adopted to search the TCDB database [5], during which pairwise and domain similarities are integrated and initial predictions are made. The phase is similar to our previous approach except that topology analysis is not included [20]. Secondly, machine learning methods are used to improve the initial predictions, during which both non-homology evidence and homology evidence from other sources are integrated, such as transmembrane segments and the consistency of TC families of the top-K nearest neighbors in TCDB [20], homologs in Pfam [9] and Gene Ontology [10] databases, and non-transporter homologs from SwissProt [21]. All forms of evidence are converted into features of a refining classifier and rules to discriminate true positives from false positives of the initial classifier are learned from some well-studied model organisms. Unlike TransAPP [8], which integrates evidence by empirical rules, TransportTP searches for discriminative rules using machine learning methods, which is more efficient and systematic, since certain types of evidence may be conflicting with each other, or may be biased in different databases, such as Pfam [9] and Gene Ontology [10]. Thus, manual handling of empirical rules are very difficult to be efficient, but machine learning methods are efficient at searching for the discriminative boundary in a large evidence space.

TransportTP was implemented as both Linux command lines and a web server. Only basic information, such as nucleotide/amino acid sequences, predictive options like training organisms and initial e-value threshold, are required by TransportTP for prediction. The predictive results are presented in a user-friendly manner through a web interface, enumerating all evidence used in decision making and providing cross-links from the evidence to related databases for further verification. Results are sortable via various criteria selected by the user, to accommodate specific interests or curation emphasis. Implementation of the system is very efficient due to the use of parallel computing algorithms among multiple CPUs in a single computer and/or computers spanning a local network.

## Results and Discussion

### Data and performance assessment

The databases for implementation and testing of TransportTP were downloaded in September 2008. The TCDB [5] for the initial classifier consisted of 5,005 transporters within 557 TC families/superfamilies, of which 173 possessed at least 5 members with corresponding HMMs. The terms of family and superfamily are alternatively used in this manuscript because they mix in the third taxonomic level of the TC system. If a superfamily exists in a hierarchical branch, the superfamily was studied rather than its constituent TC families. Pfam (ver 23.0) [9], Gene Ontology (GO) [10] and SwissProt (ver 14.2) [21] databases contained 10,340 domains, and 163,260 and 398,181 curated sequences, respectively. Eleven organisms were chosen for cross-validation of TransportTP, including seven model organisms (*Escherichia coli O157:H7 EDL933*, *Saccharomyces cerevisiae S288C*, *Drosophila melanogaster*, *Caenorhabditis elegans*, *Arabidopsis thaliana*, *Oryza sativa *and *Homo sapiens*), and four non-model organisms (*Picrophilus torridus DSM 970*, *Photobacterium profundum SS9*, *Desulfotalea psychrophila LSv54 *and *Aspergillus fumigatus*). Sequences and functional annotations of the eleven organisms were acquired from NCBI, except for *S. cerevisiae *and *O. sativa*, which were obtained from SGD ftp://ftp.yeastgenome.org/yeast/data_download/sequence/genomic_sequence/orf_protein/archive/ and JCVI ftp://ftp.jcvi.org/pub/data/Eukaryotic_Projects/o_sativa/annotation_dbs/pseudomolecules/version_5.0/ respectively. Five plant organims were chosen for case studies, including *Sorghum bicolor*, *Populus trichocarpa*, *Vitis vinifera*, *Physcomitrella patens *and *Chlamydomonas reinhardtii*. The genomic sequences of the five plant organisms were downloaded from JGI Microbial Genomics ftp://ftp.jgi-psf.org/pub/JGI_data, except for *Vitis vinifera*, which was downloaded from Genoscope http://www.genoscope.cns.fr/externe/Download/Projets/Projet_ML/data/annotation/Vitis_vinifera_peptide_v1.fa.

Three measurements were applied in the performance assessment of TransportTP. The first was recall, defined as the proportion of transporters in test sets which were exactly categorized by TransportTP with the same TC family or superfamily. The test sets were either TransportDB [8] in cross-validations or curated transporters in case studies. The second measurement was precision, defined as the proportion of predicted transporters by TransportTP which exactly matched the test sets in TC family/superfamily. The third measurement was balanced accuracy, which was the overall assessment of a single test and was defined as:(1)

These three measurements are also known as sensitivity, selectivity, and F-measure, respectively. The average value of each measurement on multiple testing organisms reflects the predictive performance of TransportTP on the specific training organisms(s). Specificity and the Receiver Operating Characteristic curve were not used in our assessment because non-transporters are much more than the transporters, making them less informative.

### Cross-validation test

The performance of TransportTP was tested by two cross-validation schemas: (1) Leave-one-in (LOI) cross-validation, i.e. choosing the proteome of one model organism for training and the proteomes of other ten organisms for testing; (2) Leave-multiple-in (LMI) cross-validation, i.e. choosing the proteomes of all seven model organisms for training and only the proteomes of the four non-model organisms for testing. Redundant protein sequences with a similarity of 40% or above were removed during the training to avoid potential overestimation of prediction accuracy. TransportDB [7,8] was used as a benchmark transporter database in the cross-validation.

The leave-one-in cross-validation intended to examine whether the classification rules learned from one model organism can effectively predict transporters in other organisms. *S. cerevisiae *(yeast) was chosen as an example organism in our analysis with e-value threshold set to 0.1, since the best predictive results were often achieved around this e-value threshold for most training organisms. The cross-validation results of the example are shown in Table 1. On average, 81.8% (recall) of the curated transporters of the ten non-yeast testing organisms in TransportDB were exactly predicted by TransportTP. The exact matches accounted for 81.8% (precision) of the predictions and corresponded to a balanced accuracy of 81.8% as well. Besides, there were 7.6% of predictions validated by a text mining program through the comparison of functional annotation along with the protein sequences (See Methods Section for details). Those predictions were not counted in the stringent assessment but they might be *bona fide *transporters excluded by TransportDB [8]. The mismatch at superfamily or family level between our predictions and TransportDB was only 0.2% among the predicted population. Only a slight difference in performance was observed between model and non-model organisms, with the balanced accuracy of 82.0% and 81.4%, average recall of 81.7% and 81.8%, and average precision of 82.2% and 81.1%, respectively. This result indicated that the over-representation of transporters of model organisms in the training datasets did not influence significantly the predictive accuracy due to efficient avoidance of overtraining by the Support Vector Machines [22] applied in the refining classifier. On the contrary, other factors such as evolutionary distance between the training organisms and the testing organisms may contribute more to the difference of predictive accuracy. The performance between model and non-model organisms training on other individual organisms also supports this hypothesis (see Additional file 1).

**Table 1:** The cross-validation results yielded by yeast.

Organism	Num of proteins	Predictions by TransportTP	Annotations in TransportDB	Matches	Mismatches	TransportDB unique	Text mining validated	Recall (%)	Precision (%)	Balanced accuracy (%)
*E. coli*	5411	589	577	456	6	115	61	79.0	77.4	78.2
*A. thaliana*	26960	1073	1278	996	1	281	38	77.9	92.8	84.7
*O. sativa*	56278	1230	1283	1061	0	222	88	82.7	86.3	84.4
*C. elegans*	20051	906	667	601	1	65	87	90.1	66.3	76.4
*D. melanogaster*	13890	663	646	535	0	111	26	82.8	80.7	81.7
*H. sapiens*	37742	1272	1466	1140	3	323	79	77.8	89.6	83.3
Average on model proteomes								81.7	82.2	82.0

*P. torridus*	1535	165	171	137	1	33	15	80.1	83.0	81.5
*P. profundum*	5489	550	580	445	4	131	35	76.7	80.9	78.8
*D. psychrophila*	3234	316	305	242	1	62	38	79.3	76.6	77.9
*A. fumigatus*	9923	671	619	563	1	55	50	91.0	83.9	87.3
Average on non-model proteomes								81.8	81.1	81.4

Average on all testing proteomes								81.7	81.8	81.8

The proteome of the yeast was used for training and the ten non-yeast proteomes were used for testing at the e-value threshold of 0.1. TransportDB was chosen for the benchmark transporter database. The "matches" column represents the number of proteins predicted by TransportTP and curated by TransportDB with the same TC family or superfamily (the third taxonomic level). The "mismatches" column corresponds to the number of proteins predicted as transporters by both methods but with conflicting TC classification. The column of "TransportDB unique" is the number of proteins annotated by TransportDB but absent in the predictions of TransportTP. The number of "text mining validated" corresponds to the number of proteins not annotated by TransportDB but predicted by TransportTP and validated by our text mining program through the functional annotations together with protein sequences.

Predictive performance differed significantly between major transporter classes. The best performance was achieved for carriers, followed by channels, and finally primary active transporters. More specifically, TransportTP achieved a balanced accuracy of 87.9% on 74 carrier families, 61.5% on 31 channel families, and 54.6% on 8 primary active transporter families that TransportDB reported on the ten non-yeast testing organisms, at the e-value threshold of 0.1 (see Additional file 2 for details). This difference in performance is likely due to factors such as distinct transporter mechanisms within transporter classes, other than difference in sequence divergence in transporter families, since the correlation between the balanced accuracy and sequence identity of the predicted families/superfamilies on the ten testing organisms was 0.23, a weak correlation.

**Figure 2:**
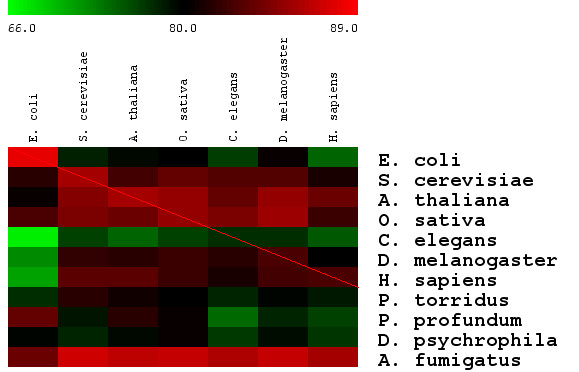
**Balanced accuracy of TransportTP in the leave-one-in cross-validation**. The proteome of one of the seven model organisms was used for training and the proteomes of ten other organisms were used for testing at the e-value threshold of 0.1. Training organisms are shown in columns and the testing organisms, in rows. Cells represent the corresponding degree of balanced accuracy in percentage, using color defined by the heat map above.

The complete results of leave-one-in cross-validation tests at the e-value threshold of 0.1 are shown in Figure 2 and Additional file 3. Although the performance varied with respect to various training and testing organisms, it can be summarized as follows: (1) the average balanced accuracy was 80.6% among 70 cross-validations (excluding self-training and testing), of which 43 (61%) cross-validations had a balanced accuracy of over 80%. If e-value thresholds were variable for specific training and testing organisms, 53 out of the 70 (76%) cross-validations achieved a best balanced accuracy of over 80%; (2) *E. coli *yielded the worse performance among the seven training model organisms because it was the only prokaryotic organism used for training. *C. elegans *had the worst testing performance for most training organisms probably due to the poor annotation of this organism, as reflected by many un-annotated proteins in NCBI; (3) The performance of a testing organism was greatly influenced by the evolutionary distance to the training organism. The diagonal elements in the figure generally served as the performance peaks since they correspond to zero evolutionary distances between training and testing organism.

The leave-multiple-in (LMI) validation results using all model organisms for training and the four non-model organisms for testing are shown in Table 2 and Additional file 4. Two fold results were observed from the comparison of this approach with leave-one-in (LOI) approach using individual model organism for training. The LMI approach achieved better balanced accuracy on the four non-model testing organisms than the LOI approach using *C. elegans*, *D. melanogaster*, *H. sapiens *and *E. coli *for training, but comparable to that using *S. cerevisiae*, *A. thaliana *and *O. sativa *for training. For example, the LMI approach achieved comparable balanced accuracy (80.2%) to that yielded by the yeast alone (81.4%) in the LOI approach. Nevertheless, the maximal loss of balanced accuracy using all model organisms for training was 3.2%, compared with any individual organism for training at the same e-value threshold, therefore, it seems to be a good trade off using all model organisms for training if no well-studied model organism exists.

**Table 2:** The cross-validation results yielded by all model organisms.

Organism	Num of proteins	Predictions by TransportTP	Annotations in TransportDB	Matches	Mismatches	TransportDB unique	Text mining validated	Recall (%)	Precision (%)	Balanced accuracy (%)
*P. torridus*	1535	169	171	134	1	36	16	78.4	79.3	78.8
*P. profundum*	5489	603	580	472	4	104	41	81.4	78.3	79.8
*D. psychrophila*	3234	327	305	246	1	58	37	80.7	75.2	77.9
*A. fumigatus*	9923	705	619	556	1	62	52	89.8	78.9	84.0
Average on non-model proteomes								82.6	78.0	80.2

The proteomes of seven model organisms were used for training and proteomes of four non-model organisms were used for testing at the e-value threshold of 0.1. TransportDB was chosen for the benchmark transporter database. The definition of the columns can be referred to Table 1.

### Comparative study of performance

The performance of TransportTP was also studied by comparing it with other approaches, using at a broad range of e-value thresholds between 10 and 1e-50, to reveal the comprehensive characteristics of TransportTP and alternative strategies.

**Figure 3:**
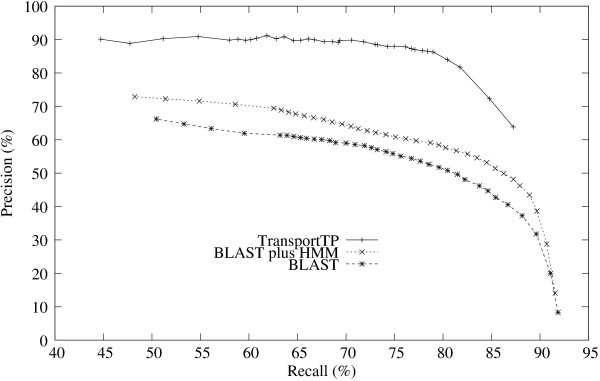
**Precision versus recall of TransportTP compared with alternative approaches**. Precision versus recall was comparatively studied among TransportTP, BLAST plus HMM, and BLAST alone. The yeast proteome was used for training and the proteomes of ten other organisms was used for testing at e-value thresholds between 10 and 1e-50 (e-values not shown). Precision and recall were averaged on the ten non-yeast testing organisms.

**Figure 4:**
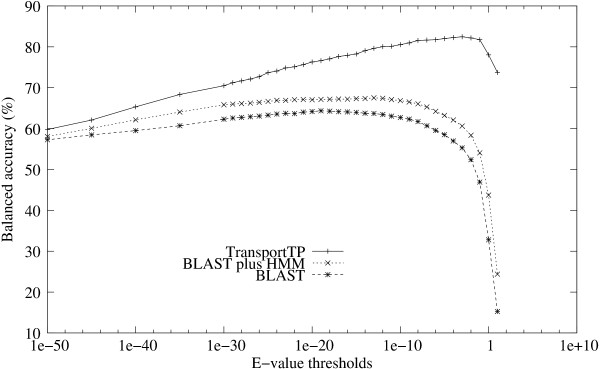
**Balanced accuracy of TransportTP compared with alternative approaches**. Balanced accuracy was comparatively studied among TransportTP, BLAST plus HMM, and BLAST alone. The yeast proteome was used for training and the proteomes of ten other organisms was used for testing.

Firstly, TransportTP was compared with BLAST search, the approach used widely by biologists, and the integration of BLAST and HMM, the approach applied in our initial classifier. *S. cerevisiae *(yeast) was again chosen as an example training organism for analysis. The comparative results are shown in Figure 3 and Figure 4. TransportTP significantly outperformed both BLAST search and BLAST plus HMM, as measured by precision versus recall over a wide range of E-value thresholds (Figure 3). Superior performance of TransportTP versus BLAST plus HMM demonstrates the value of integrating various transporter-related evidence in the refining classifier of our method. Figure 3 also demonstrates the value of integrating HMM models with BLAST search in the initial classifier of TransportTP since BLAST plus HMM was always superior to BLAST alone.

The advantages of TransportTP are further demonstrated in Figure 4, which shows the average balanced accuracy of the ten non-yeast testing organisms versus e-value thresholds adopted in the initial classifier. TransportTP outperformed both BLAST plus HMM and the BLAST search alone in balanced accuracy, especially at commonly used e-value thresholds. At the threshold e-value 10, TransportTP outperformed BLAST plus HMM by 49.3%. The great increase of balanced accuracy resulted from an increase of precision from 14.1% to 63.8% along with a relatively small decrease of recall from 91.6% to 87.2% (corresponded to the rightmost points of the two curves in Figure 3). At this high e-value threshold, most transporters were covered in the initial classifier, making it possible for the refining classifier to discriminate the true positives from the false positives. At very low e-value thresholds, many true positives are excluded and few false positives are included, limiting the power of the refining classifier to generate effective discriminative rules.

TransportTP was also compared with other reported methods. Compared with our previous work [20], which was similar to our initial classifier but included the TMS filtering, TransportTP achieved a better balanced accuracy of 81.8% compared with 67.0% in the previous approach. More specifically, TransportTP significantly improved the precision from 55.9% to 81.8%, with a slight sacrifice in recall from 83.6% to 81.8% (For fairness, validations through the text mining program in the previous approach were excluded in this comparison). Compared with SVMPort [15], TransportTP achieved better performance in recall, precision and most importantly, the much larger coverage of TC families, since SVMPort only achieved an average recall of 81.0% and an average precision of 26.1% among five TC superfamilies and three families in an independent evaluation set.

### Case studies

The performance of TransportTP was investigated further using sequences from five model plant oraganisms, based on the availability of whole proteome and their evolutionary divergence. *Sorghum bicolor *(sorghum), *Populus trichocarpa *(poplar) and *Vitis vinifera *(grape) are important agricultural organisms. *Physcomitrella patens *(moss) is a simplest plant model for plant functional evolutionary studies, and *Chlamydomonas reinhardtii *(green alga) is a single-cell green alga organism, representing the group from which land plants evolved. These organisms span a large part of the plant family tree, from single-celled to vascular plants, including monocots to dicots, so that the performance on these organisms should reflect the performance of TransportTP on plant transporters in general. Finally, although most of these organisms were recently sequenced, they lack good annotation for membrane transporters [8].

The performance of TransportTP on plant organisms was evaluated via the comparison of the predictive results of the program with the manual curation by our biologists. Automatic transporter prediction was learned from the model plant organisms *Arabidopsis thaliana *at an e-value threshold of 10, to cover as many transporters as possible. Manual transporter curation was carried out via human review of all transporter-related evidence from candidate proteins, specifically transmembrane proteins and homologs of TCDB transporters. The evidence included all data utilized by TransportTP, such as predicted membrane topology, presence of homologs in TCDB [5], conserved Pfam domains [9], Gene Ontology terms [10], and presence of homologs in SwissProt database [21]. In addition, evidence which was difficult to manage by automatic prediction, such as annotation of homology hits in the NCBI NR-Refseq database [23], were also reviewed. Curated putative membrane transporters were organized into confidence levels based on types of confidence for the classification. Level one corresponded to the highest confidence, in which almost all expected pieces of evidence for a transporter superfamily/family supported the classification. Level two corresponded to a moderate confidence, where a minor piece of evidence was conflicting or missing (such as a little bit short of protein length). Level three corresponded to the lowest confidence level, in which multiple types of evidence or an important evidence were missing (such as lack of characteristically conserved domains and/or too small protein length), raising doubts about the transporter functionality or gene annotation. The union of level-one and level-two confidence levels was considered as a benchmark for manual curation of membrane transporters, while the level-three was only taken as reference and disregarded in further analysis. The detailed results are hosted at http://bioinfo3.noble.org/transporter/model.htm.

The comparative results for manual curation versus automatic prediction of transporters for the five plant organisms are shown in Table 3. On average, 74.6% of putative transporters curated manually for the four plant organisms, excluding the green alga, were exactly predicted by TransportTP. The exact matches accounted for 73.4% of the automatic predictions. Another 8.6% of automatic predictions exactly matched manual curation with level-three confidence, which may also include some *bona fide *transporters.

**Table 3:** Case study results of TransportTP on five plant organisms.

Organism	Curated	Predicted	Matches	Recall (%)	Precision (%)	Potential rate(%)
*S. Bicolor*	1918	1960	1485	77.4	75.8	7.7
*P. trichocarpa*	2512	2889	1936	77.1	67.0	14.4
*V. vinifera*	2188	2002	1540	70.4	76.9	5.5
*P. patens*	1388	1380	1019	73.4	73.8	6.8
average				74.6	73.4	8.6

*C. reinhardtii*	979	770	554	56.6	71.9	7.7

The manual curation and automatic prediction by TransportTP were compared in five plant organisms at the e-value threshold of 10. The column of curated is the number of transporters manually curated with confidence level 1 or level 2. The fourth column corresponds to the number of predictions matched the curation with confidence levels 1 and 2. The last column is the proportion of predictions matched the curation with confidence level 3.

Predictions of the green alga were less successful presumably because of the evolutionary distance from Arabidopsis, the training organism. Nevertheless, TransportTP still achieved a recall of 56.6% with respect to the manual curation approach and a precision of 72.0% on the automatic prediction. Compared with TransportDB which includes *P. patens *and *C. reinhardtii*, TransportTP achieved a recall of 77.6% and a precision of 78.6% on *P. patens *at an e-value threshold of 1, and achieved a recall of 71.5% and a precision of 72.8% on *C. reinhardtii *at an e-value threshold of 0.01 (see Additional file 5 for details). The results demonstrate a solid performance of TransportTP in predicting transporters on plant organisms using a model plant proteome for training.

Further investigation of the comparative results revealed that the confidence levels of manual curation were correlated with their recall of TransportTP on the groups. Specifically, 79.6%, 49.2% and 19.4% of curated transporters in confidence levels 1, 2 and 3 were exactly predicted by TransportTP, respectively, on the five plant organisms explored (details not shown). The low recall made by TransportTP for confidence level 3 does not deny the effectiveness of TransportTP, since the manual curation could not reliably assert this group of potential transporters into superfamilies/families, indicating major problems for classification of these proteins.

## Discussion

We adopted stringent assessment in the cross-validation of TransportTP, where only predictions matching with curated transporters in TransportDB were counted in correct ones during the training and the testing. However, TransportDB manually excluded some categories of transporters [8], resulting in these transporter categories incorrectly trained and undetectable in the testing for any genome being annotated. Therefore, if the coverage of the benchmark database improves, the predictive performance will be further increased, both for the cross-validation and for case studies.

We did not adopt the standard k-folds or leave-one-out cross-validations but instead, used leave-one-in strategy, because the underlying transporter mechanisms in different organisms may be distinct although they share very large similarity. For example, the distribution of transporter families and the gating mechanisms of transporters are likely to be different between prokaryotic and eukaryotic organisms [24]. Thus, the combination of multiple organisms for training a classification model may not always increase the predictive accuracy, as shown in Additional file 4.

We did not adopt traditional Support Vector Machine (SVM) but used an ensemble and balanced SVMs in the refining classifier, to handle the unbalanced data produced by the initial classifier. The comparative results of using traditional Support Vector Machine (SVM) [22] without the ensemble and balanced technology are shown in Additional file 6. Although TransportTP with and without the ensemble of balanced SVMs achieved almost equivalent balanced accuracy with respect to the same e-value threshold used in the initial classifier, TransportTP outperformed traditional SVMs measured by precision versus recall, especially when the initial predictions were highly imbalanced with true and false positives. Similarly, the ensemble and balanced techniques brought improvement to precision versus recall for decision trees like CART [25]. However, the balanced random forest of decision trees [26] was not adopted in TransportTP because of its over-training on model organisms and unstable performance, as shown in Additional file 6.

Despite of the effectiveness and efficiency, some potential pitfalls of TransportTP exist. Firstly, although non-homology pieces of evidence contribute significantly to the performance, sequence homology still plays a nontrivial rule in the inference of novel transporters. If some proteins are very similar to transporters but in fact they are not transporters, for example, receptors or sensors, which evolved from transporters but diverged functionally since the evolutionary split [27], they may be annotated as transporters incorrectly. Fortunately, the inclusion of characteristic features such as presence of conserved domains and number of transmembrane segments in our method may help distinguishing these proteins from the transporters in a great extent.

Secondly, TransportTP targets on individual transporters rather than multimeric transporters, which are complex functional structures assembled from products of multiple genes. For example, human TAP transporters dimers [28], while glutamate transporters are trimers [29]. For those multimeric transporters, each polypeptide chain is classified individually into a transporter family according to the characteristics assigned for the chain, even when some other chains are not found to complete the transporter function. We opted to offer the possibility for the researchers to find the whole multimeric transporter structure, which would require further analysis such as examining the interactions among the chains and functional evolution of each chain.

Thirdly, TransportTP generally relies on complete protein sequences for classification, since partial sequences will introduce doubts on functionality of the gene/peptide, thus being confined to lower confidence levels. When handling partial sequences such as some ESTs, the classification will depend on the key regions of the proteins available for classification, such as unique conserved domains and transmembrane segments. In a nutshell, the system is built to rely on whole protein sequences, but can be useful for partial sequences, with loss of robustness.

## Conclusions

In summary, the effectiveness and the utility of TransportTP has been demonstrated in detail through cross-validation between model and non-model organisms, comparative study with alternative strategies in the cross-validation, and the case studies of plant organisms. TransportTP will be of importance for researchers working on annotation of newly assembled genomes, especially eukaryotic genomes, and will probably be used as an additional step for classification of genes/proteins that cannot be clearly classified as transporters by using existing database resources. The approach of TransportTP may be of interest for improvement of broad classification tasks, showing how new classification rules can be extracted from sequences through combination of homology and non-homology evidence.

## Methods

### Preprocessor

The framework of TransportTP is shown in Figure 1. It consisted of two components, a pre-processor and a predictor, and two interfaces, i.e. a web interface and a command line interface. The predictor was further divided into two phases: an initial classifier followed by a refining classifier.

The pre-processor constructed transporter-related databases and models for the predictor. A transporter-related Gene Ontology database [10] was constructed through the exaction of a subgraph rooted at the term GO:0022857, which corresponded to transmembrane transporter activities. The subgraph contained 561 transporter terms and associated 5,393 transporter sequences. Meanwhile, a transporter-related Pfam domain database was constructed via: (1) all cross-links between Pfam domains and TC families embedded in the Pfam database [9], (2) additional mapping between TC families and Pfam domains, constructed via all-versus-all HMM search [9] between TC sequences and Pfam domains, where a TC family was linked to a Pfam domain if at least a proportion (50% in implementation) of members in the TC family contained the Pfam domain, (3) other manually curated mappings. Consequently, 487 Pfam domains were mapped to 320 TC families or superfamilies (see Additional file 7). The construction of transporter-related databases was necessary because the inclusion of non-transporter terms or domains had little contribution to the predictive performance while significantly increased the computation cost. Two kinds of models were built for the predictors. An HMM model was constructed for each TC family/superfamily in TCDB [6] for the initial classifier if having enough cardinality, through the SAM program due to no pre-alignment requirement of member proteins [30]. A classification model was constructed for the refining classifier from some well-studied model organisms during which the initial classifier was invoked but the refining classifier was not involved, to avoid potential circling.

### Initial classifier

The initial classifier of TransportTP started categorization of unknown proteins among hundreds of TC families in the TCDB and an exceptional group, i.e. non-transporter, using sequence homology based search. The BLAST and HMM e-value scores of an unknown protein, corresponding to the pairwise and family similarity with respect to the TCDB database, were combined, since this combination improves the predictive accuracy [20,31]. Specifically, the two similarity scores were weighted by the square root of their product, to trade off the two similarities. The transporter in the TCDB database which had the smallest weighted score was said to be the nearest neighbor of the unknown protein. Correspondingly, the attached TC family/superfamily of the nearest neighbor was initially predicted as the family or superfamily of the unknown protein if the weighted score was below a user-specified threshold; otherwise, it was categorized as a non-transporter. To handle the case that many transporters in TCDB might not be associated with HMM scores due to the limited member of transporters in their TC families, thus impossible to develop HMM models, a weighted score may be directly compared with an unweighted score, for the comparability arising from their roughly equivalent implications. The formalization of the initial classifier is in Equation 2 and more details can be referred to our previous paper [20].(2)

In equation 2, *p *is an unknown protein for prediction, *s*(*p*) is the weighted score, *f*(*p*) is the classification function and *λ *is the threshold to discriminate transporters from non-transporters; *q*_*ij *_is the *jth *transporter in the *ith *TC family *F*_*i*_, *blast*(*p, q*_*ij*_) is the BLAST e-value between the protein *p *and the transporter *q*_*ij *_and the *hmm*(*p, MDL*_*i*_) is the HMM score of protein *p *on the transporter family *F*_*i*_, where *MDL*_*i *_is the HMM model built for the family, |*F*_*i*_| is the cardinality of family *F*_*i*_, *κ *is the threshold of family cardinality for construction of HMM models and *τ *is the threshold of BLAST search. The three cases in calculating *s*(*p*) for transporter *q*_*ij *_corresponds to 1) both BLAST and HMM scores being available, 2) only HMM e-value being available and 3) only BLAST e-value being available.

### Refining classifier

The refining classifier was to distinguish false positives from true positives generated by the initial classifier, because numerous false positives may be generated due to the lack of negative features of non-transporters in the TCDB database. The discriminative rules between false positive and true positives were learned from some well-studied model organisms based on a variety of transporter-related features. The excluded non-transporters by the initial classifier were not further refined because the initial classifier was designed to cover most of true positives, thus the small number of false negatives, i.e. missed transporters, would not influence the overall predictive accuracy while greatly increasing the efficiency. Seven categories of features for initially categorized proteins were extracted from transporter-related databases and the input sequences. The first was the basic homology scores to TCDB database generated by the initial classifier, specifically the BLAST and HMM e-value scores, calculated by Tera-BLASTP [4] and SAM [30], respectively. The second category of features was the initially categorized transporter classes, such as channels, carriers, or primary active transporters, and the sizes of the initially categorized transporter families, since the size may impact the quality of the homology inference. The family size was transformed logarithmically to avoid potential dominative impact.

The third category of features for initially categorized proteins was the number of transmembrane segments (TMS) in the proteins and in the initially categorized TC families. The number of TMS within most TC families are conserved throughout evolution, due to specific functional requirements [24]. Therefore, beyond the TMS number as a basic feature of an unknown protein, a z-score measurement [32] was applied as another feature of the protein, to approximately estimate the match extent between an unknown protein and the predicted TC family in TMS number. The z-score was calculated as follows:(3)

where *tms*(*p*) was the number of TMS of a protein *p*, taken as the maximum calculated by HMMTOP [33] and TMPRED [34], *π *and *σ *were the mean and standard deviation of TMS of the initially categorized TC family *F *of the protein *p *and *ξ *was a tiny constant to prevent the denominator of being zero.

The fourth category of features for an initially categorized protein was the consistency of TC families among the top-K homologs (K is a small constant [35]) of the protein in the TCDB, evaluated by the proportion of the top-K homologs possessing the same TC families as that of the top homolog of the protein in the TCDB. High consistency is a very positive sign for potential transporters. The feature was also amended as an additional feature for the cases where the cardinalities of the predicted families were smaller than the constant K, in which K was adjusted to the cardinality of the initially categorized family to capture the maximal possibility of consistency.

The fifth category of features for an initially categorized protein was the occurrence of transporter-related Pfam domains, calculated by HMMER [9] with user specified e-value threshold. The occurrence of transporter-related domains provided an important clue for the potential transporters, especially for families showing characteristically conserved domains. Two Pfam features of an unknown protein were extracted for the refining classifier. The first feature was the best e-value score among the transporter-related Pfam domains and the second was whether the occurred Pfam domains coincided with the initial categorized TC family of the unknown protein, through checking the mapping between Pfam domains and TC families established in the preprocessor.

The sixth category of features for an initially categorized protein was the hits of transporter-related Gene Ontology terms [10], via the BLAST search of the protein against the transporters attached in transporter-related GO terms [4]. A hit of transporter-related GO terms was a positive sign of potential transporters and the best e-value score among the hit transporter sequences in the transporter-related GO database was extracted as a GO feature of the unknown protein for the refining classifier. Whether one of the hit GO terms coincided with its initially categorized TC family in function was considered as another feature of the unknown protein. To simplify the situation where each transporter logically belonged to a branch of GO terms, TransportTP only counted the directly belonging GO terms as hit terms and searched for the functional coincidence between these hit terms and the initially predicted TC families, using a text mining program we developed. The text mining program justified the consistency between two descriptions if enough significant overlapping words were found. Obviously insignificant English words were filtered but compatible words were counted, based on a series of compatibility rules generated on the basis of biological activity; for example: the abbreviation K+ was compatible with words such as potassium, ions and metal. The last category of features was the negative information from UniProt/SwissProt [21]. If the nearest neighbor of an unknown protein in SwissProt was more similar to the protein than the nearest neighbor in TCDB and its function in SwissProt implied a non-transporter function, such as a transcription factor, the initial categorization of the protein based on TCDB was more likely to be a false positive. Based on this principle, the existence of this kind of confliction between the nearest neighbors in TCDB and SwissProt was extracted as a feature of the protein for the refining classifier. The list of keywords chosen for detection of non-transporter functions is shown in Additional file 8.

The classification value of an initially categorized protein for the refining classifier was defined as whether the initial classifier categorized the protein with a correct TC family. A false classification value meant that the protein should be removed from the final predictions. A true classification value of a training protein in the refining classifier was determined by the match with a curated transporter at family or superfamily level in TransportDB [7,8]. TransportDB was chosen as a benchmark database because the transporters collected in this database were curated by biologists, hence the data therein was relatively reliable.

Support Vector Machine (SVM) in the Weka package [36] was adopted in the training and testing of the refining classifier because it could optimally determine the decision boundary of two classes with the least over-training [22]. An ensemble of balanced SVM approach was developed to specifically handle the highly imbalanced data in the refining classifier at most e-value thresholds. All instances of the minor classes were selected but only a part of instances in the majority class with the amount equal to the cardinality of the minor class were randomly selected without replacement for each SVM classifier. The number of SVMs in the ensemble was proportional to the imbalanced ratio, which was:(4)

where *N*_*major *_and *N*_*minor *_were the number of instances in the majority and minority classes, respectively, and *β *was a constant. If *β *was large enough, being 10 in our implementation, most instances in the majority class would have a chance to be explored. The ensemble of balanced SVMs vote by their confidences to decide the final predicted classification value for an unknown protein. The stochastic and voting strategies have been similarly applied in handling imbalanced data and proved to be effective [37,38].

## Availability and requirements

Project name: A two-phase transporter categorization system

Project home page: http://bioinfo3.noble.org/transporter.

Operating system: Platform independent.

Program language: C++, Perl and JSP.

Other requirement: Java application server.

Any restrictions to use by non-academics: Licence needed for non-academic use and source code available for academic purpose upon request.

## Authors' contributions

HL designed and implemented the algorithms. VB and MU curated transporters in plant organisms. PZ conceived and directed the project. All authors read and approved the final manuscript.

## Supplementary Material

Additional file 1 **Comparative performance of TransportTP on non-model and model organisms**. PDF displaying relative balanced accuracy, recall and precision of TransportTP on non-model organisms subtracted that on model organisms in leave-one-in cross-validations. Click here for file

Additional file 2 **Performance of TransportTP on transporter families covered by TransportDB**. This PDF table displays recall, precision and balanced accuracy of TransportTP on transporter families covered by TransportDB, using the proteome of yeast for training, the proteomes of ten other organisms for testing, and 0.1 as e-value threshold. Click here for file

Additional file 3 **Complete validation results of TransportTP on seven training model-organisms**. PDF displaying complete balanced accuracy, recall and precision of TransportTP in leave-on-in cross-validations at e-value threshold of 0.1, where one of the proteomes of the seven model organisms was used for training and the proteomes of ten other organisms were used for testing. Click here for file

Additional file 4 **Comparative results of TransportTP on non-model organisms using leave-multiple-in versus leave-one-in cross-validations**. PDF displaying relative balanced accuracy, recall and precision of TransportTP on non-model organisms yielded by leave-multiple-in cross-validations subtracted that of leave-one-in cross-validations. Click here for file

Additional file 5 **Validation results of TransportTP on P. patens and C. reinhardtii using TransportDB as the benchmark database**. This PDF displays validation results of TransportTP on *P. patens *and *C. reinhardtii *using arabidopsis for training and e-value thresholds between 10 and 0.00001 for homology search, and TransportDB for the benchmark database. Click here for file

Additional file 6 **Comparative performance of TransportTP with four alternative refining classifiers**. This PDF displays the comparative recall versus precision, and balanced accuracy versus e-value thresholds among TransportTP with ensemble of balanced SVMs, balanced random forest, traditional SVM, and decision tree J48. Click here for file

Additional file 7 **Mapping of Pfam domains to Transporter Classification (TC) families/superfamilies**. This PDF table displays the mapping of Pfam domains to Transporter Classification families or superfamilies, using 1) cross-links in Pfam database, 2) automatic mapping between Pfam domains and TC families/superfamilies and 3) manual curation. Click here for file

Additional file 8 **The keywords adopted in the text-mining program to detect non-transporter functions in Swiss-Prot database**. This PDF displays the keywords adopted in the text-mining program to detect non-transporter functions hit by unknown proteins in Swiss-prot Database. Click here for file

## References

[B1] Sakmann B Neher E Patch clamp techniques for studying ionic channels in excitable membranes Annu Rev Physiol 1984 46 455 472 10.1146/annurev.ph.46.030184.002323 6143532

[B2] Hsu L Chiou T Chen L Bush D Cloning a plant amino acid transporter by functional complementation of a yeast amino acid transport mutant Proc Natl Acad Sci USA 1993 90 7441 7445 10.1073/pnas.90.16.7441 8356039 PMC47157

[B3] Kuze K Graves P Leahy A Wilson P Stuhlmann H You G Heterologous expression and functional characterization of a mouse renal organic anion transporter in mammalian cells J Biol Chem 1999 274 1519 1524 10.1074/jbc.274.3.1519 9880528

[B4] Altschul S Gish W Miller W Myers E Lipman D Basic local alignment search tool J Mol Biol 1990 215 403 410 2231712 10.1016/S0022-2836(05)80360-2

[B5] Saier MJ Tran C Barabote R TCDB: the Transporter Classification Database for membrane transport protein analyses and information Nucleic Acids Res 2006 34 Database D181 D186 10.1093/nar/gkj001 16381841 PMC1334385

[B6] Saier M Yen M Noto K Tamang D Elkan C The Transporter Classification Database: recent advances Nucleic Acids Res 2009 37 Database D274 278 10.1093/nar/gkn862 19022853 PMC2686586

[B7] Ren Q Kang K Paulsen I TransportDB: a relational database of cellular membrane transport systems Nucleic Acids Res 2004 32 Database D284 D288 10.1093/nar/gkh016 14681414 PMC308751

[B8] Ren Q Chen K Paulsen I TransportDB: a comprehensive database resource for cytoplasmic membrane transport systems and outer membrane channels Nucleic Acids Res 2007 35 Database D274 D279 10.1093/nar/gkl925 17135193 PMC1747178

[B9] Sonnhammer E Eddy S Durbin R Pfam: a comprehensive database of protein domain families based on seed alignments Proteins 1997 28 405 420 10.1002/(SICI)1097-0134(199707)28:3<405::AID-PROT10>3.0.CO;2-L 9223186

[B10] Ashburner M Ball C Blake J Gene ontology: tool for the unification of biology. The Gene Ontology Consortium Nat Genet 2000 25 25 29 10.1038/75556 10802651 PMC3037419

[B11] Devos D Valencia A Intrinsic errors in genome annotation Trends Genet 2001 17 429 431 10.1016/S0168-9525(01)02348-4 11485799

[B12] Koski L Golding G The closest BLAST hit is often not the nearest neighbor J Mol Evol 2001 52 540 542 11443357 10.1007/s002390010184

[B13] Doolittle R Similar amino acid sequences: chance or common ancestry? Science 1981 214 149 159 10.1126/science.7280687 7280687

[B14] Bejerano G Yonam G Variations on probabilistic suffix trees: statistical modeling and prediction of protein families Bioinformatics 2001 17 23 43 10.1093/bioinformatics/17.1.23 11222260

[B15] Lin H Han L Cai C Ji Z Chen Y Prediction of transporter family from protein sequence by support vector machine approach Proteins 2006 62 218 231 10.1002/prot.20605 16287089

[B16] Dibrov P Fliegel L Comparative molecular analysis of Na+/H+ exchangers: a unified model for Na+/H+ antiport? FEBS Lett 1998 424 1 5 10.1016/S0014-5793(98)00119-7 9537504

[B17] Heil B Ludwig J Lichtenberg-Frate H Lengauer T Computational recognition of potassium channel sequences Bioinformatics 2006 22 1562 1568 10.1093/bioinformatics/btl132 16595554

[B18] Gromiha M Yabuki Y Functional discrimination of membrane proteins using machine learning techniques BMC Bioinformatics 2008 9 135 10.1186/1471-2105-9-135 18312695 PMC2375119

[B19] Lee M Jeong C Kim D Predicting and improving the protein sequence alignment quality by support vector regression BMC Bioinformatics 2007 8 471 10.1186/1471-2105-8-471 18053160 PMC2222655

[B20] Li H Dai X Zhao X A nearest neighbor approach for automated transporter prediction and categorization from protein sequences Bioinformatics 2008 24 1129 1136 10.1093/bioinformatics/btn099 18337257

[B21] Apweiler R Functional information in SWISS-PROT: the basis for large-scale characterisation of protein sequences Brief Bioinform 2001 2 9 18 10.1093/bib/2.1.9 11465066

[B22] Platt JC Advances in kernel methods: support vector learning, Cambridge, MA, USA: MIT Press 1999 chap. Fast training of support vector machines using sequential minimal optimization 185 208

[B23] Pruitt K Tatusova T Maglott D NCBI Reference Sequence (RefSeq): a curated non-redundant sequence database of genomes, transcripts and proteins Nucl Acids Res 2005 33 suppl 1 D501 504 15608248 10.1093/nar/gki025 PMC539979

[B24] Saier MJ A functional-phylogenetic classification system for transmembrane solute transporters Microbiol Mol Biol Rev 2000 64 354 411 10.1128/MMBR.64.2.354-411.2000 10839820 PMC98997

[B25] Quinlan R C4.5: Programs for Machine Learning 1993 San Mateo, CA: Morgan Kaufmann Publishers

[B26] Breiman L Random Forests Machine Learning 2001 45 5 32 10.1023/A:1010933404324

[B27] Boles E Andre B Role of transporter-like sensors in glucose and amino acid signalling in yeast Top Curr Genet 2004 9 121 153

[B28] Abele R Tampe R Function of the transport complex TAP in cellular immune recognition Biochimica et Biophysica Acta (BBA) - Biomembranes 1999 1461 2 405 419 10.1016/S0005-2736(99)00171-6 10581370

[B29] Yernool D Boudker O Jin Y Gouaux E Structure of a glutamate transporter homologue from Pyrococcus horikoshii Nature 431 811 818 10.1038/nature03018 15483603

[B30] Krogh A Brown M Mian IS Sjolander K Haussler D Hidden Markov models in computational biology: Applications to protein modeling Journal of Molecular Biology 1994 235 1501 1531 10.1006/jmbi.1994.1104 8107089

[B31] Alam I Dress A Rehmsmeier M Fuellen G Comparative homology agreement search: An effective combination of homology-search methods Proc Natl Acad Sci USA 2004 101 13814 13819 10.1073/pnas.0405612101 15367730 PMC518839

[B32] Atteson K Calculating the exact probability of language-like patterns in biomolecular sequences Proceedings of the sixth International Conference on Intelligent Systems for Molecular Biology (ISMB), Canada 1998 17 24 9783205

[B33] Tusnady G Simon I The HMMTOP transmembrane topology prediction server Bioinformatics 2001 17 849 50 10.1093/bioinformatics/17.9.849 11590105

[B34] Hofmann K Stoffel W TMbase - A database of membrane spanning proteins segments Biol Chem 1993 374 166

[B35] Horton P Nakai K Better prediction of protein cellular localization sites with the k nearest neighbors classifier Proc Int Conf Intell Syst Mol Biol, Halkidiki, Greece 1997 5 147 152 9322029

[B36] Witten I Frank E Data mining: practical machine learning tools and techniques with Java implementations ACM SIGMOD Record 2002 31 76 77 10.1145/507338.507355

[B37] Akbani R Kwek S Japkowicz N Applying Support Vector Machines to Imbalanced Datasets ECML 2004 39 50

[B38] Wang BX Japkowicz N Boosting Support Vector Machines for Imbalanced Data Sets ISMIS 2008 38 47

