# Supplementary material for: TransportTP: A two-phase classification approach for membrane transporter prediction and characterization
Source: BMC Bioinformatics. 2009 Dec 14;10:418. doi: 10.1186/1471-2105-10-418 (PMC3087344; doi:10.1186/1471-2105-10-418)
Supplement: Additional file 1 — Comparative performance of TransportTP on non-model and model organisms. PDF displaying relative balanced accuracy, recall and precision of TransportTP on non-model organisms subtracted that on model organisms in leave-one-in cross-validations. [file 1471-2105-10-418-S1.PDF]

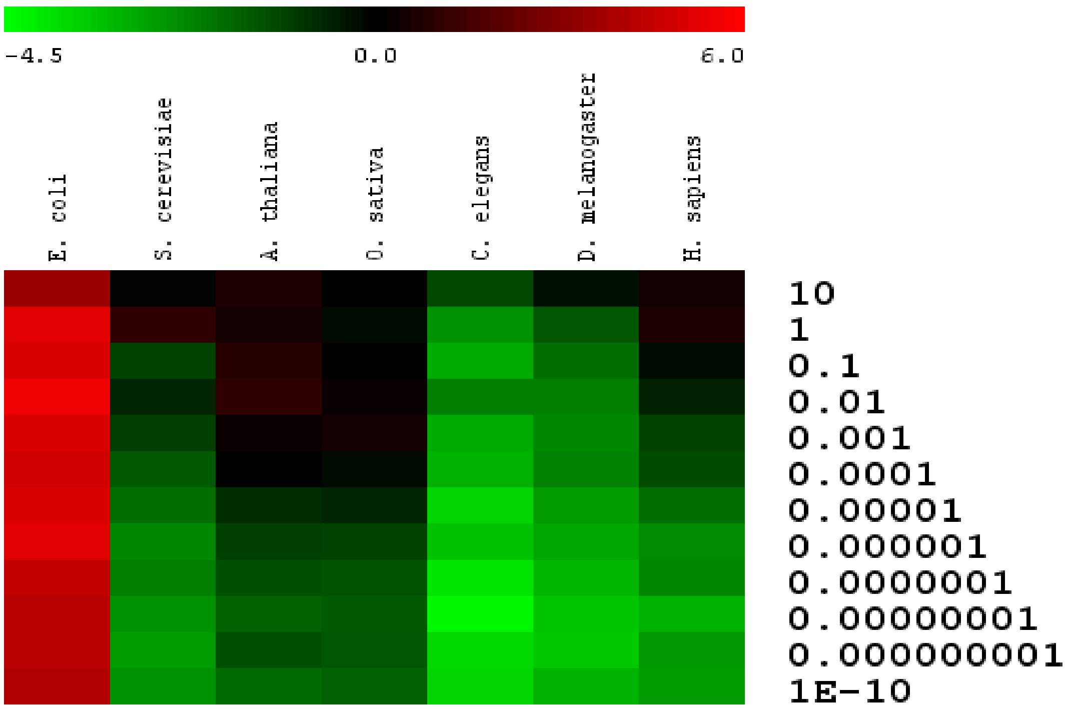

Figure S1_a. The average balanced accuracy of TransportTP on the four non-model organisms subtracts that on the six testing model organisms (excluded the training organism) using different model organisms for training at various e-value thresholds. The difference of balanced accuracy is shown in percentage scale[1]. The figure indicates that 1) Model organisms did not always achieve better balanced accuracy than non-model organisms in the testing; 2) The balanced accuracy depends on both the distance between the testing organism and training organism and the e-value thresholds used in the initial classifier. The less distance and larger e-value threshold led to the better balanced accuracy on non-model organisms.

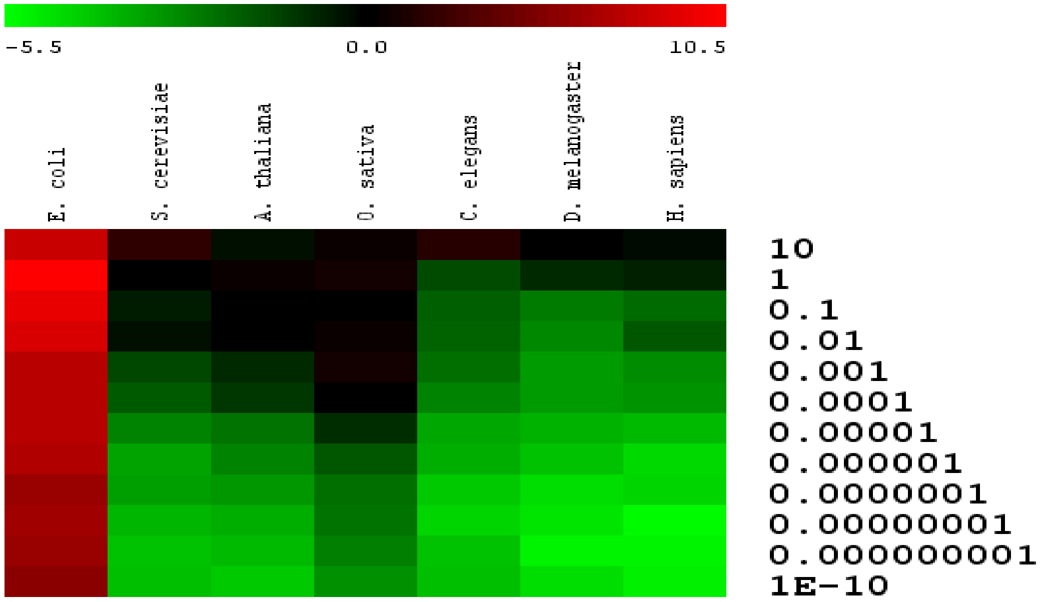

[1] The scales of recall, precision and balanced accuracy are in percentage in all tables and figures of the supplementary data, without specifically description.

Figure S1_b. The recall of TransportTP on the non-model organisms subtracts that on the six testing model organisms (excluded the training organism) using different model organisms for training at various e-value thresholds. The difference of recall is in percentage scale. The figure indicates that the recall of TransportTP on the non-model organisms is generally worse than that on the six testing model organisms, except that yielded by *E.coli*. This may be due to the larger evolutionary distance from the training *organisms* to the four testing non-model organisms than that of the other six testing model organisms, so that less homologs can be found by the initial classifier in the non-model organisms, except that yielded by *E. coli*.

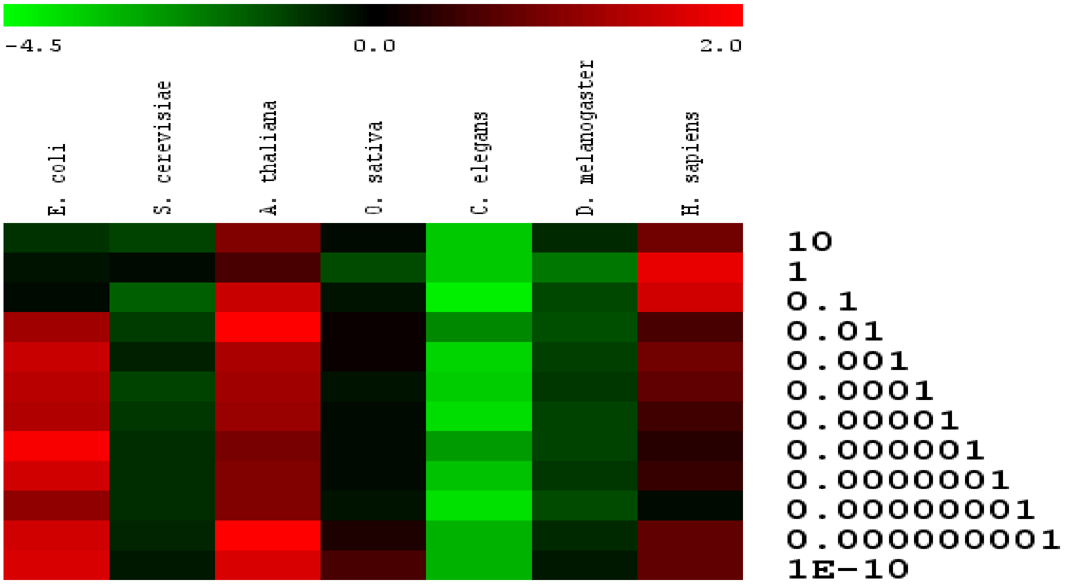

Figure S1_c. The precision of TransportTP on the four non-model organisms subtracts that on the six testing model organisms (excluded the training organism) using different model organisms for training at various e-value thresholds. The difference of precision is shown in percentage scale. The figure indicates that the individual model organisms generally achieve equivalent or better precision on non-model organisms than on the six testing model organisms (except the two animal organisms and the yeast), probably due to the low recall on the non-model organisms or smaller evolutationary distance (for *E. coli*).
