# Supplementary material for: TransportTP: A two-phase classification approach for membrane transporter prediction and characterization
Source: BMC Bioinformatics. 2009 Dec 14;10:418. doi: 10.1186/1471-2105-10-418 (PMC3087344; doi:10.1186/1471-2105-10-418)
Supplement: Additional file 2 — Performance of TransportTP on transporter families covered by TransportDB. This PDF table displays recall, precision and balanced accuracy of TransportTP on transporter families covered by TransportDB, using the proteome of yeast for training, the proteomes of ten other organisms for testing, and 0.1 as e-value threshold. [file 1471-2105-10-418-S2.PDF]

Family id	Family description	Family size	Num of TMS_	Dev of TMS	TDB reported	Predictions	Matches	Recall (%)	Precion (%)	Balanced accuracy (%)
1.A.1.	The Voltage-gated Ion Channel (VIC) Superfamily	115	8.47	5.75	327	556	420	100.00	75.54	86.07
1.A.10.	The Glutamate-gated Ion Channel (GIC) Family of Neurotransmitter Receptors	13	4.54	1.28	106	131	114	100.00	87.02	93.06
1.A.11.	The Ammonia Transporter Channel (Amt) Family	19	10.58	2.54	43	43	43	100.00	100.00	100.00
1.A.12.	The Intracellular Chloride Channel (CLIC) Family	7	0.86	0.35	16	24	12	75.00	50.00	60.00
1.A.13.	The Epithelial Chloride Channel (E-ClC) Family	2	1.5	0.5	4	9	3	75.00	33.33	46.15
1.A.15.	The Non-selective Cation Channel-2 (NSCC2) Family	2	3	1	6	0	0	0.00	0.00	0.00
1.A.2.	Inward Rectifier K+ Channel (IRK-C) Family	10	3.1	0.83	33	35	33	100.00	94.29	97.06
1.A.20.	The BCL2/Adenovirus E1B-interacting Protein 3 (BNip3) Family	2	1	0	2	0	0	0.00	0.00	0.00
1.A.21.	The Bcl-2 (Bcl-2) Family	2	2	0	22	2	2	9.09	100.00	16.67
1.A.22.	The Large Conductance Mechanosensitive Ion Channel (MscL) Family	3	2.67	0.47	2	1	1	50.00	100.00	66.67
1.A.23.	The Small Conductance Mechanosensitive Ion Channel (MscS) Family	7	6.71	3.19	35	36	34	97.14	94.44	95.77
1.A.24.	The Gap Junction-forming Connexin (Connexin) Family	8	4	0	28	28	28	100.00	100.00	100.00
1.A.25.	The Gap Junction-forming Innexin (Innexin) Family	8	4.62	0.7	33	45	35	100.00	77.78	87.50
1.A.27.	The Phospholemman (PLM) Family	6	1.83	0.37	15	9	9	60.00	100.00	75.00
1.A.28.	The Urea Transporter (UT) Family	4	9.75	0.83	2	2	2	100.00	100.00	100.00
1.A.3.	The Ryanodine-Inositol 1,4,5-triphosphate Receptor Ca2+ Channel (RIR-CaC) Family	3	7.33	1.89	14	41	14	100.00	34.15	50.91
1.A.31.	The Annexin (Annexin) Family	4	0	0	49	0	0	0.00	0.00	0.00
1.A.35.	The CorA Metal Ion Transporter (MIT) Family	7	2.14	0.35	30	24	22	73.33	91.67	81.48
1.A.4.	The Transient Receptor Potential Ca2+ Channel (TRP-CC) Family	36	7.44	1.3	45	146	46	100.00	31.51	47.92
1.A.46.	The Anion Channel-forming Bestrophin (Bestrophin) Family	4	5	1.22	30	35	30	100.00	85.71	92.31

1.A.48.	The Anion Channel Tweety (Tweety) Family	4	5.5	0.5	5	9	5	100.00	55.56	71.43
1.A.5.	The Polycystin Cation Channel (PCC) Family	6	7.67	1.8	19	18	14	73.68	77.78	75.68
1.A.50.	The Phospholamban (Ca2+-channel and Ca2+-ATPase Regulator) (PLB) Family	2	1	0	2	0	0	0.00	0.00	0.00
1.A.52.	The Ca2+ Release-activated Ca2+ (CRAC) Channel (CRAC-C) Family	3	3.33	0.94	4	0	0	0.00	0.00	0.00
1.A.54.	The Presenilin ER Ca2+ Leak Channel (Presenilin) Family	3	9	0	5	0	0	0.00	0.00	0.00
1.A.56.	The Copper Transporter (Ctr) Family	13	2.62	0.49	24	27	23	95.83	85.19	90.20
1.A.6.	The Epithelial Na+ Channel (ENaC) Family	24	2	0.58	60	78	60	100.00	76.92	86.96
1.A.62.	The Homotrimeric Cation Channel (TRIC) Family	2	6.5	0.5	2	0	0	0.00	0.00	0.00
1.A.7.	ATP-gated Cation Channel (ACC) Family	5	2	0.63	14	14	14	100.00	100.00	100.00
1.A.8.	The Major Intrinsic Protein (MIP) Family	42	6.14	0.47	113	141	134	100.00	95.04	97.45
1.A.9.	The Neurotransmitter Receptor, Cys loop, Ligand-gated Ion Channel (LIC) Family	22	4.05	1.22	151	222	178	100.00	80.18	89.00
2.A.1.	The Major Facilitator Superfamily (MFS)	312	12	1.73	1118	1466	1261	100.00	86.02	92.48
2.A.12.	The ATP:ADP Antiporter (AAA) Family	12	11.83	0.55	4	10	5	100.00	50.00	66.67
2.A.13.	The C4-Dicarboxylate Uptake (Dcu) Family	3	11	0	9	9	9	100.00	100.00	100.00
2.A.14.	The Lactate Permease (LctP) Family	3	17	1.41	6	6	6	100.00	100.00	100.00
2.A.15.	The Betaine/Carnitine/Choline Transporter (BCCT) Family	6	12	0	13	13	13	100.00	100.00	100.00
2.A.16.	The Telurite-resistance/Dicarboxylate Transporter (TDT) Family	6	9	1.41	26	27	26	100.00	96.30	98.11
2.A.17.	The Proton-dependent Oligopeptide Transporter (POT) Family	15	11.4	2.94	167	205	196	100.00	95.61	97.76
2.A.18.	The Amino Acid/Auxin Permease (AAAP) Family	26	10.73	0.86	171	203	196	100.00	96.55	98.25
2.A.19.	The Ca2+:Cation Antiporter (CaCA) Family	18	11.11	1.1	74	94	83	100.00	88.30	93.79
2.A.2.	The Glycoside-Pentoside-Hexuronide (GPH):Cation Symporter Family	15	11.73	0.77	40	42	42	100.00	100.00	100.00
2.A.20.	The Inorganic Phosphate Transporter (PiT) Family	9	10.67	0.82	18	21	20	100.00	95.24	97.56
2.A.21.	The Solute:Sodium	21	13.43	0.66	55	58	57	100.00	98.28	99.13

	Symporter (SSS) Family									
2.A.22.	The Neurotransmitter:Sodium Symporter (NSS) Family	27	11.44	1.71	61	85	79	100.00	92.94	96.34
2.A.23.	The Dicarboxylate/Amino Acid:Cation (Na+ or H+) Symporter (DAACS) Family	13	9.38	1.73	31	35	35	100.00	100.00	100.00
2.A.24.	The 2-Hydroxycarboxylate Transporter (2-HCT) Family	8	12.25	0.83	1	1	1	100.00	100.00	100.00
2.A.25.	The Alanine or Glycine:Cation Symporter (AGCS) Family	3	10.33	0.94	12	12	12	100.00	100.00	100.00
2.A.26.	The Branched Chain Amino Acid:Cation Symporter (LIVCS) Family	4	11.75	0.43	6	6	6	100.00	100.00	100.00
2.A.27.	The Glutamate:Na+ Symporter (ESS) Family	2	11	1	3	4	3	100.00	75.00	85.71
2.A.28.	The Bile Acid:Na+ Symporter (BASS) Family	4	9	0	25	28	24	96.00	85.71	90.57
2.A.29.	The Mitochondrial Carrier (MC) Family	72	4.42	1.35	298	389	358	100.00	92.03	95.85
2.A.3.	The Amino Acid-Polyamine-Organocation (APC) Family	77	12.13	0.83	173	196	192	100.00	97.96	98.97
2.A.30.	The Cation-Chloride Cotransporter (CCC) Family	10	11.6	0.66	29	39	36	100.00	92.31	96.00
2.A.31.	The Anion Exchanger (AE) Family	11	11.45	1.56	32	49	49	100.00	100.00	100.00
2.A.33.	The NhaA Na+:H+ Antiporter (NhaA) Family	2	10	0	3	3	3	100.00	100.00	100.00
2.A.34.	The NhaB Na+:H+ Antiporter (NhaB) Family	2	11.5	0.5	2	2	2	100.00	100.00	100.00
2.A.35.	The NhaC Na+:H+ Antiporter (NhaC) Family	6	12.17	1.07	8	8	8	100.00	100.00	100.00
2.A.36.	The Monovalent Cation:Proton Antiporter-1 (CPA1) Family	25	12.4	1.02	59	77	74	100.00	96.10	98.01
2.A.37.	The Monovalent Cation:Proton Antiporter-2 (CPA2) Family	13	10.85	4.24	70	79	76	100.00	96.20	98.06
2.A.38.	The K+ Transporter (Trk) Family	20	7.65	4.33	18	18	17	94.44	94.44	94.44
2.A.39.	The Nucleobase:Cation Symporter-1 (NCS1) Family	12	12.17	0.37	13	13	12	92.31	92.31	92.31
2.A.4.	The Cation Diffusion Facilitator (CDF) Family	21	6.86	3.38	67	94	83	100.00	88.30	93.79
2.A.40.	The Nucleobase:Cation Symporter-2 (NCS2) Family	13	12.38	1	63	73	71	100.00	97.26	98.61
2.A.41.	The Concentrative Nucleoside Transporter	11	10.82	3.49	16	19	19	100.00	100.00	100.00

	(CNT) Family									
2.A.42.	The Hydroxy/Aromatic Amino Acid Permease (HAAAP) Family	6	11.17	0.37	24	28	28	100.00	100.00	100.00
2.A.43.	The Lysosomal Cystine Transporter (LCT) Family	3	6.67	0.47	12	26	15	100.00	57.69	73.17
2.A.44.	The Formate-Nitrite Transporter (FNT) Family	3	6.67	0.94	7	6	6	85.71	100.00	92.31
2.A.45.	The Arsenite-Antimonite (ArsB) Efflux Family	3	11.67	1.7	12	9	8	66.67	88.89	76.19
2.A.47.	The Divalent Anion:Na+ Symporter (DASS) Family	20	12.4	1.28	38	43	38	100.00	88.37	93.83
2.A.48.	The Reduced Folate Carrier (RFC) Family	4	11.5	0.87	10	9	9	90.00	100.00	94.74
2.A.49.	The Chloride Carrier/Channel (ClC) Family	17	11.06	1.06	48	64	64	100.00	100.00	100.00
2.A.5.	The Zinc (Zn2+)-Iron (Fe2+) Permease (ZIP) Family	19	7.58	0.88	70	80	57	81.43	71.25	76.00
2.A.50.	The Glycerol Uptake (GUP) Family	2	11.5	0.5	5	7	4	80.00	57.14	66.67
2.A.51.	The Chromate Ion Transporter (CHR) Family	3	11.67	0.47	2	2	2	100.00	100.00	100.00
2.A.52.	The Ni2+-Co2+ Transporter (NiCoT) Family	8	6.38	1.22	5	2	1	20.00	50.00	28.57
2.A.53.	The Sulfate Permease (SulP) Family	25	11.4	1.55	81	100	97	100.00	97.00	98.48
2.A.54.	The Mitochondrial Tricarboxylate Carrier (MTC) Family	2	5	0	13	18	16	100.00	88.89	94.12
2.A.55.	The Metal Ion (Mn2+-iron) Transporter (Nramp) Family	8	11.12	0.6	30	44	43	100.00	97.73	98.85
2.A.56.	The Tripartite ATP-independent Periplasmic Transporter (TRAP-T) Family	23	6.22	4.91	49	38	38	77.55	100.00	87.36
2.A.57.	The Equilibrative Nucleoside Transporter (ENT) Family	15	10.67	0.79	38	42	39	100.00	92.86	96.30
2.A.58.	The Phosphate:Na+ Symporter (PNaS) Family	4	10	1.58	7	9	7	100.00	77.78	87.50
2.A.59.	The Arsenical Resistance-3 (ACR3) Family	2	10	0	5	5	5	100.00	100.00	100.00
2.A.6.	The Resistance-Nodulation-Cell Division (RND) Superfamily	96	6.78	5.22	94	125	83	88.30	66.40	75.80
2.A.60.	The Organo Anion Transporter (OAT) Family	14	11.71	0.7	29	42	33	100.00	78.57	88.00
2.A.62.	The NhaD Na+:H+ Antiporter (NhaD) Family	2	12.5	1.5	7	7	7	100.00	100.00	100.00
2.A.64.	The Twin Arginine Targeting (Tat) Family	11	2.91	2.35	21	18	17	80.95	94.44	87.18

2.A.66.	The Multidrug/Oligosaccharidyl-lipid/Polysaccharide (MOP) Flippase Superfamily	44	12.23	1.15	151	156	150	99.34	96.15	97.72
2.A.67.	The Oligopeptide Transporter (OPT) Family	10	15.5	1.43	55	64	64	100.00	100.00	100.00
2.A.68.	The p-Aminobenzoyl-glutamate Transporter (AbgT) Family	2	12.5	0.5	4	4	4	100.00	100.00	100.00
2.A.69.	The Auxin Efflux Carrier (AEC) Family	6	10	0	40	33	30	75.00	90.91	82.19
2.A.7.	The Drug/Metabolite Transporter (DMT) Superfamily	74	8.31	2.38	389	367	345	88.69	94.01	91.27
2.A.71.	The Folate-Biopterin Transporter (FBT) Family	8	12.88	0.93	17	19	19	100.00	100.00	100.00
2.A.72.	The K+ Uptake Permease (KUP) Family	7	13	0.53	41	66	64	100.00	96.97	98.46
2.A.74.	The 4 TMS Multidrug Endosomal Transporter (MET) Family	2	4.5	0.5	3	6	3	100.00	50.00	66.67
2.A.75.	The L-Lysine Exporter (LysE) Family	2	6	0	3	3	3	100.00	100.00	100.00
2.A.76.	The Resistance to Homoserine/Threonine (RhtB) Family	5	6	0	25	24	24	96.00	100.00	97.96
2.A.78.	The Branched Chain Amino Acid Exporter (LIV-E) Family	6	4.67	1.11	2	2	2	100.00	100.00	100.00
2.A.8.	The Gluconate:H+ Symporter (GntP) Family	6	11.33	0.94	7	4	4	57.14	100.00	72.73
2.A.80.	The Tricarboxylate Transporter (TTT) Family	3	6.33	4.78	8	4	3	37.50	75.00	50.00
2.A.82.	The Organic Solute Transporter (OST) Family	4	4.25	2.77	2	0	0	0.00	0.00	0.00
2.A.85.	The Aromatic Acid Exporter (ArAE) Family	9	7.22	4.08	38	2	2	5.26	100.00	10.00
2.A.89.	The Vacuolar Iron Transporter (VIT) Family	4	5	0	9	8	1	11.11	12.50	11.76
2.A.9.	The Cytochrome Oxidase Biogenesis (Oxa1) Family	6	4.83	1.07	19	23	21	100.00	91.30	95.45
2.A.90.	The Vitamin A Receptor/Transporter (STRA6) Family	2	11	0	1	1	1	100.00	100.00	100.00
2.A.92.	The Choline Transporter -Like (CTL) Family	2	10	1	12	8	3	25.00	37.50	30.00
3.A.1.	The ATP-binding Cassette (ABC) Superfamily	876	4.31	3.92	1158	1214	1098	94.82	90.44	92.58
3.A.10.	The H+-translocating Pyrophosphatase (H+-PPase) Family	6	16.33	0.47	10	15	15	100.00	100.00	100.00
3.A.2.	The H+- or Na+-translocating F-type, V-type and A-type ATPase (F-ATPase) Superfamily	74	1.3	2.25	274	154	140	51.09	90.91	65.42

3.A.3.	The P-type ATPase (P-ATPase) Superfamily	111	8.67	2.57	244	330	286	100.00	86.67	92.86
3.A.4.	The Arsenite-Antimonite (ArsAB) Efflux Family	2	7	6	12	2	1	8.33	50.00	14.29
3.A.5.	The General Secretory Pathway (Sec) Family	42	3.64	4.23	67	52	30	44.78	57.69	50.42
3.A.8.	The Mitochondrial Protein Translocase (MPT) Family	24	1.29	1.24	112	13	13	11.61	100.00	20.80
3.A.9.	The Chloroplast Envelope Protein Translocase (CEPT or Tic-Toc) Family	10	1.6	1.36	19	0	0	0.00	0.00	0.00
