# Supplementary material for: TransportTP: A two-phase classification approach for membrane transporter prediction and characterization
Source: BMC Bioinformatics. 2009 Dec 14;10:418. doi: 10.1186/1471-2105-10-418 (PMC3087344; doi:10.1186/1471-2105-10-418)
Supplement: Additional file 3 — Complete validation results of TransportTP on seven training model-organisms. PDF displaying complete balanced accuracy, recall and precision of TransportTP in leave-on-in cross-validations at e-value threshold of 0.1, where one of the proteomes of the seven model organisms was used for training and the proteomes of ten other organisms were used for testing. [file 1471-2105-10-418-S3.PDF]

Table A3_a. The balanced accuracy of TransportTP on the eleven organisms at the e-value threshold of 0.1 using different model organisms for training. The numbers correspond to Figure 2 of the manuscript.

Testing\training	*E. coli*	*S. cerevisiae*	*A. thaliana*	*O. sativa*	*C. elegans*	*D. melanogaster*	*H. sapiens*
E. coli	88.03	78.22	79.56	80.17	76.67	80.58	74.35
S. cerevisiae	81.49	85.84	82.42	83.51	83.13	82.89	81.13
A. thaliana	80.57	84.73	85.87	85.29	83.60	85.34	83.89
O. sativa	82.80	84.44	83.89	85.20	84.40	85.62	82.03
C. elegans	66.96	76.42	74.46	76.48	77.46	77.45	75.12
D. melanogaster	72.53	81.74	81.53	82.13	81.65	82.60	79.91
H. sapiens	71.18	83.27	83.27	82.04	80.97	82.50	82.70
P. torridus	77.48	81.55	80.70	80.24	77.85	79.63	78.47
P. profundum	83.58	78.76	81.52	80.39	74.11	77.83	76.48
D. psychrophila	79.59	77.94	79.56	80.52	76.84	79.18	77.14
A. fumigatus	83.82	87.28	86.62	86.89	86.19	86.92	85.83

Table S3_b. The 'best' balanced accuracy of TransportTP on the eleven organisms at e-value thresholds between 10 and 1e-10 using different model organisms for training.

Testing\training	*E. coli*	*S. cerevisiae*	*A. thaliana*	*O. sativa*	*C. elegans*	*D. melanogaster*	*H. sapiens*
E. coli	88.03	81.43	82.79	82.57	76.67	81.52	80.25
S. cerevisiae	82.21	85.84	83.95	83.87	83.34	83.78	83.26
A. thaliana	80.79	84.73	86.57	85.30	84.08	85.34	85.79
O. sativa	82.80	84.92	85.99	86.23	84.40	86.26	84.90
C. elegans	67.35	78.72	76.63	77.11	77.95	78.29	78.14
D. melanogaster	73.12	82.86	83.36	82.87	81.74	83.63	83.59
H. sapiens	72.79	83.36	84.22	83.33	81.21	83.34	85.29
P. torridus	79.07	81.55	81.79	80.98	80.12	80.34	80.23
P. profundum	83.58	79.19	82.41	81.16	75.08	78.05	78.35
D. psychrophila	79.74	80.34	81.68	81.77	76.87	79.18	81.00
A. fumigatus	84.34	88.03	88.42	88.14	86.75	87.72	87.95

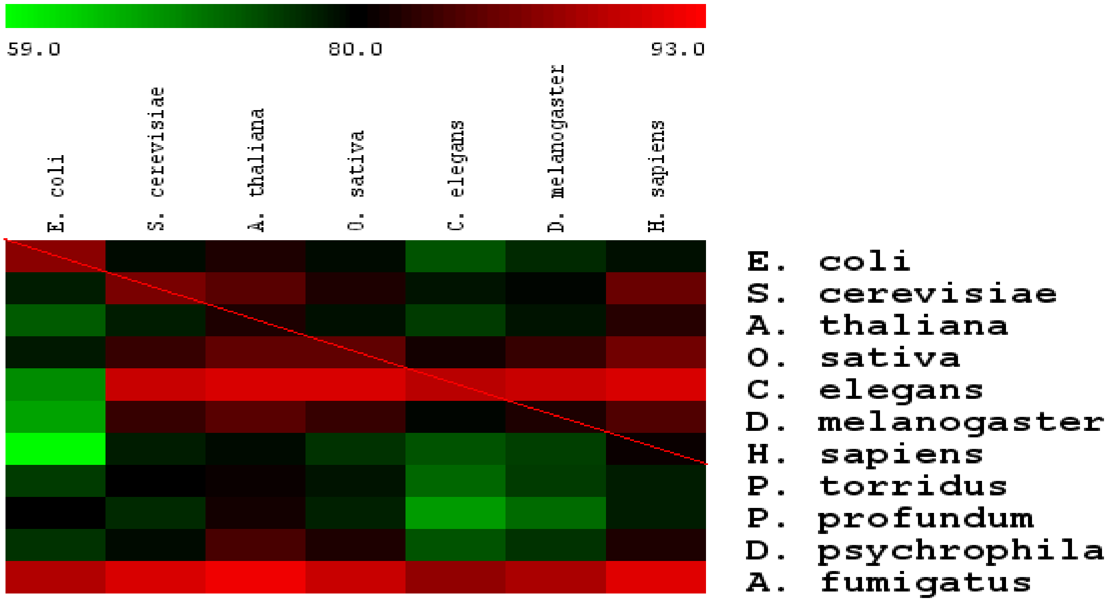

Figure S3_c. The recall of TransportTP (in percentage) on the eleven organisms at the e-value threshold of 0.1 using seven model organisms for training. Training organisms are shown in columns and testing organisms, in rows. TransportTP achieved second worst recall when using *C. elegans* for training but achieved the second best recall when using *C. elegans* for testing. The outlier of *C. elegans* coincides with the fact that many proteins of this organism in NCBI remain to be annotated. With a few exceptions, the diagonal elements, marked with a red line,and corresponded to identical testing and training organisms, had the maximal recall among all the training organisms, i.e. the maximum in the row. The recall generally decreased with the distance between testing and training organisms.

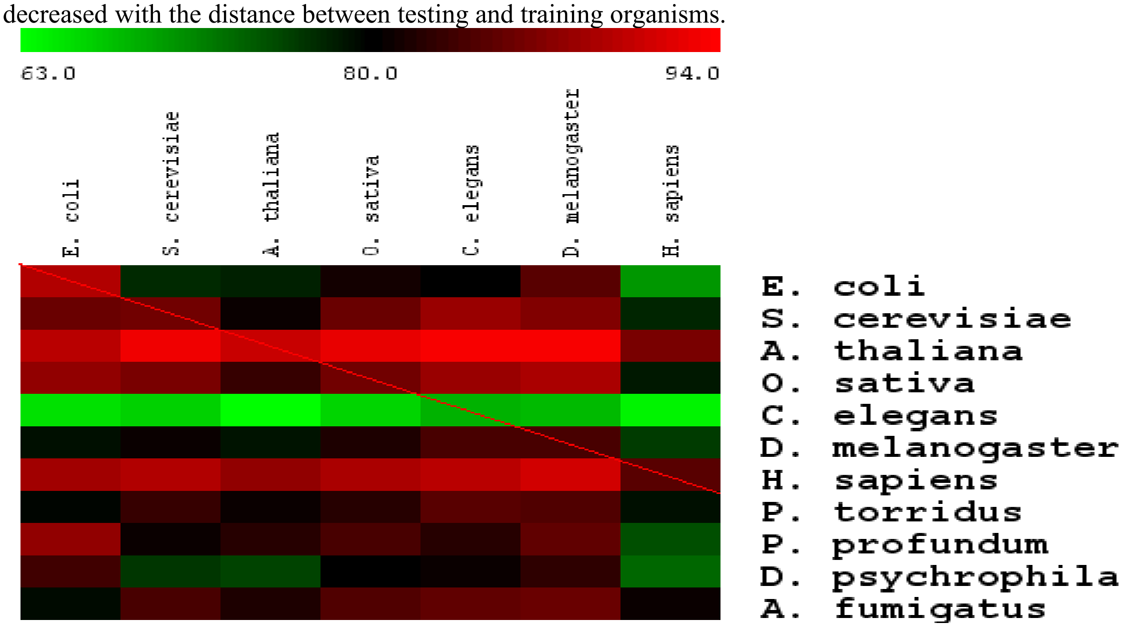

Figure S3_d. The precision of TransportTP (in percentage) on the eleven organisms at e-value threshold 0.1 using the seven model organisms for training. Training organisms are shown in columns and testing organisms, in rows. *S. cerevisiae*, *A. thaliana* and *H. sapiens* achieved the top precisions while *C. elegans* achieved the worst precision in the testing, probably due to the intensive study on the mostly important model organisms against somehow less profound study on *C. elegans*. With few exceptions, the diagonal elements, marked with a red line and corresponded to the same testing and training organism, achieved the best precision among all the training organisms, i.e. the maximum in the row.
