# Supplementary material for: TransportTP: A two-phase classification approach for membrane transporter prediction and characterization
Source: BMC Bioinformatics. 2009 Dec 14;10:418. doi: 10.1186/1471-2105-10-418 (PMC3087344; doi:10.1186/1471-2105-10-418)
Supplement: Additional file 4 — Comparative results of TransportTP on non-model organisms using leave-multiple-in versus leave-one-in cross-validations. PDF displaying relative balanced accuracy, recall and precision of TransportTP on non-model organisms yielded by leave-multiple-in cross-validations subtracted that of leave-one-in cross-validations. [file 1471-2105-10-418-S4.PDF]

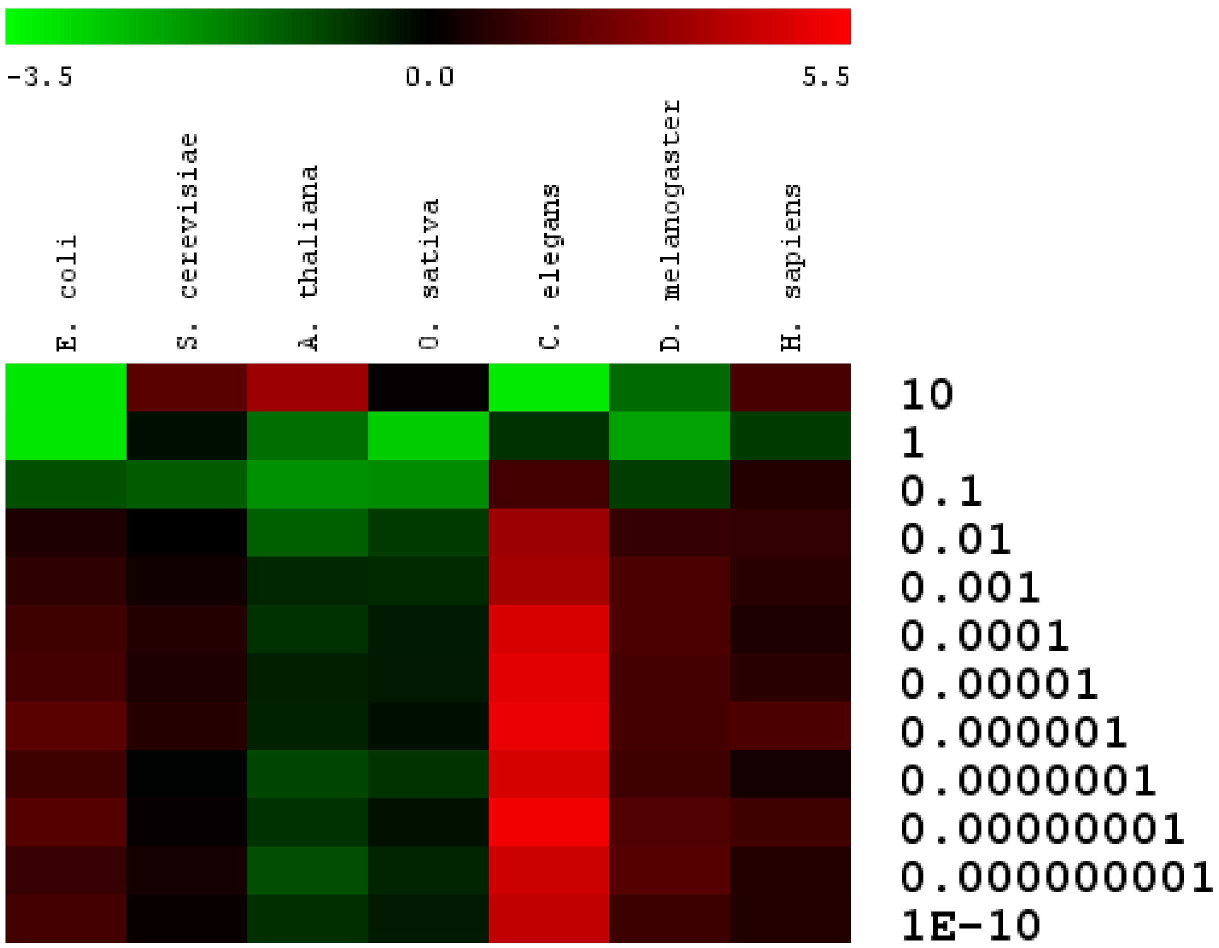

Figure S4_a. The balanced accuracy of TransportTP (in percentage) on the four non-model organisms using all model organisms for training subtracts that used individual model organisms for training at various e-value thresholds. The balanced accuracy on non-model organisms using all model organisms for training was better than that yielded by *C. elegans, D.melanogaster*, *H. sapiens* and *E.coli* but comparable to *S.cerevisiae*, *A. thaliana* and *O. sativa* at e-value thresholds less than 0.1. It was generally worse than any organism for training at e-value thresholds between 10 and 0.1 probably due to the combination of many false positives of the initial classifier from different organisms, which prevented TransportTP handling the noise data very effectively.

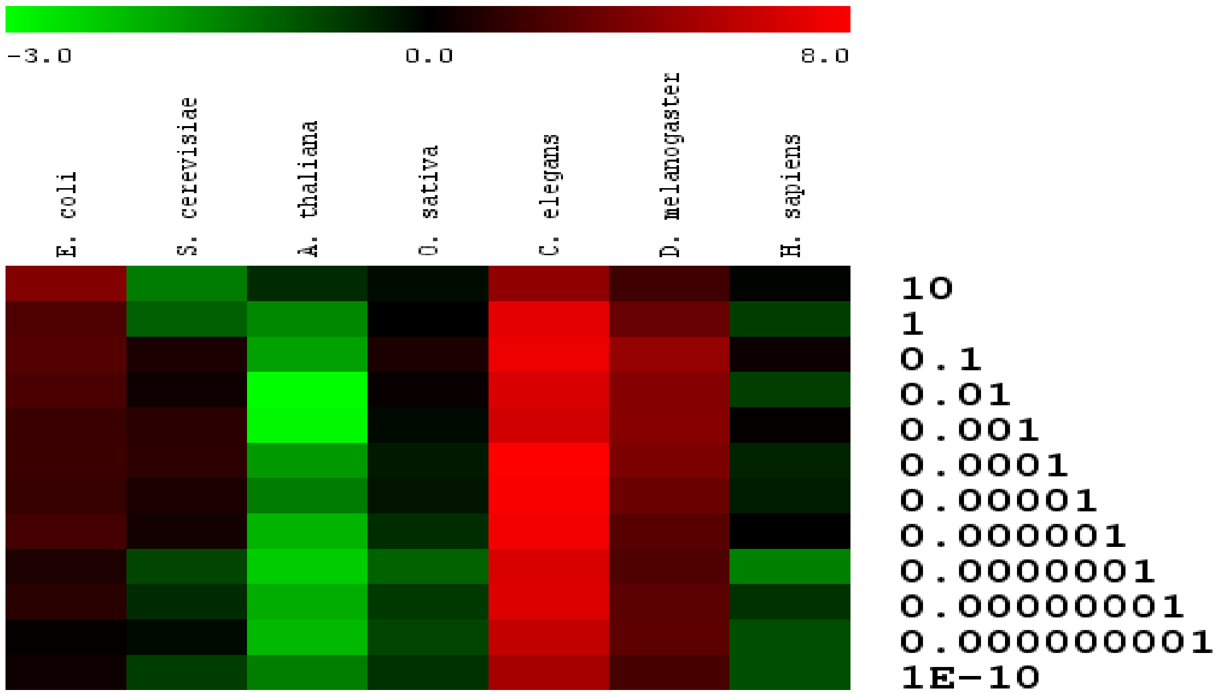

Figure S4_b. The recall of TransportTP (in percentage) on the four non-model organisms using all model organisms for training subtracts that used individual model organisms for training at various e-value thresholds. The recall of TransportTP using all model organisms for training was better than that yielded by *C. elegans*, *D. melanogaster* and *E. coli,* but worse than that yielded by *A. thaliana* , and were comparable to that yielded by *S. cerevisiae, O. sativa* and *H. sapiens*.

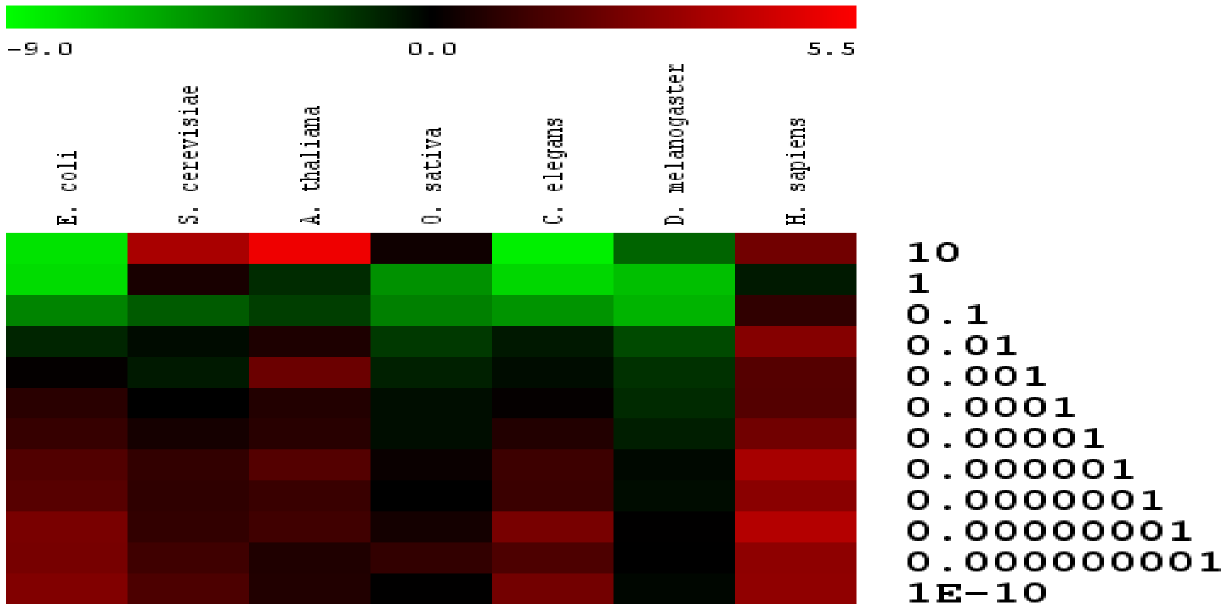

Figure S4_c. The precision of TransportTP (in percentage) on the four non-model organisms using all model organisms for training subtracts that used individual model organisms for training at various e-value thresholds. The precision of TransportTP on the non-model organisms using all model organisms for training was better than that yielded by *H. sapiens* but worse than that yielded by *D. melanogaster*, and comparable to or better than that yielded by the other five organisms at e-value threshold less than 0.1. It was generally worse than any model organism at e-value thresholds between 10 and 0.1 because many false positives were generated at the range of e-value thresholds.
