# Supplementary material for: TransportTP: A two-phase classification approach for membrane transporter prediction and characterization
Source: BMC Bioinformatics. 2009 Dec 14;10:418. doi: 10.1186/1471-2105-10-418 (PMC3087344; doi:10.1186/1471-2105-10-418)
Supplement: Additional file 5 — Validation results of TransportTP on P. patens and C. reinhardtii using TransportDB as the benchmark database. This PDF displays validation results of TransportTP on P. patens and C. reinhardtii using arabidopsis for training and e-value thresholds between 10 and 0.00001 for homology search, and TransportDB for the benchmark database. [file 1471-2105-10-418-S5.PDF]

Table S5_a. The validation results of *P.patens* using TransportDB as benchmark at e-value thresholds between 10 and 0.00001.

Annotations in TransportDB	Predictions by TransportTP	E-value Threshold	Matches	Recall (%)	Precision (%)
1050	1380	10	872	83.05	63.19
1050	1037	1	815	77.62	78.59
1050	940	0.1	797	75.90	84.79
1050	921	0.01	798	76.00	86.64
1050	902	0.001	793	75.52	87.92
1050	849	0.0001	761	72.48	89.63
1050	840	0.00001	745	70.95	89.76

Table S5_b. The validation results of *C. reinhardtii* using TransportDB as benchmark at e-value thresholds between 10 and 0.00001.

Annotations by TransportDB	Predictions by TransportTP	E-value Threshold	Matches	Recall (%)	Precision (%)
519	770	10	394	75.92	51.17
519	601	1	386	74.37	64.23
519	533	0.1	373	71.87	69.98
519	510	0.01	371	71.48	72.75
519	489	0.001	367	70.71	75.05
519	451	0.0001	347	66.86	76.94
519	425	0.00001	340	65.51	80.00
