# Supplementary material for: TransportTP: A two-phase classification approach for membrane transporter prediction and characterization
Source: BMC Bioinformatics. 2009 Dec 14;10:418. doi: 10.1186/1471-2105-10-418 (PMC3087344; doi:10.1186/1471-2105-10-418)
Supplement: Additional file 6 — Comparative performance of TransportTP with four alternative refining classifiers. This PDF displays the comparative recall versus precision, and balanced accuracy versus e-value thresholds among TransportTP with ensemble of balanced SVMs, balanced random forest, traditional SVM, and decision tree J48. [file 1471-2105-10-418-S6.PDF]

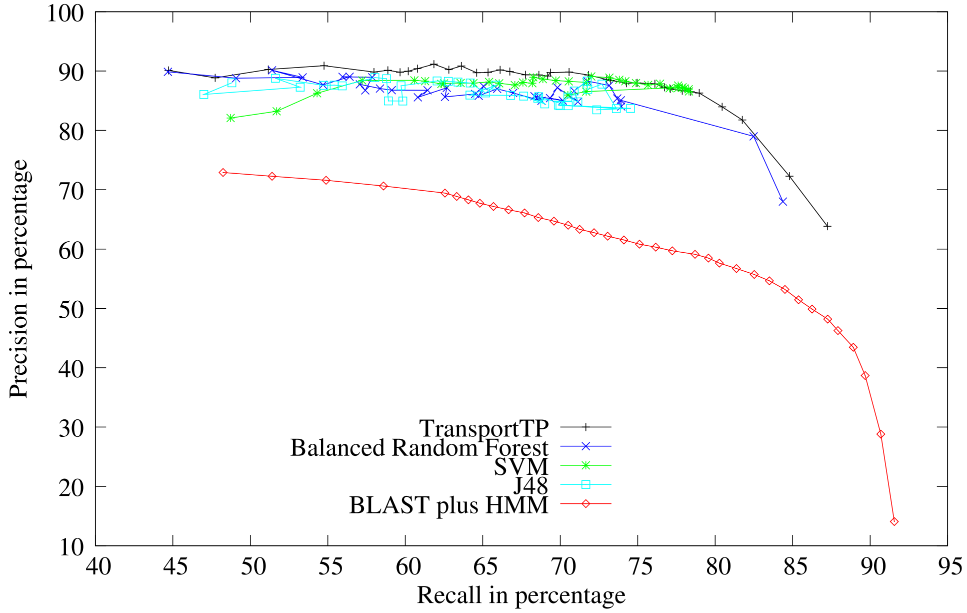

Figure 1: Precision versus recall of TransportTP with ensemble of balanced SVMs, balanced random forest, traditional SVM and decision tree J48, respectively. TransportTP without the refining classifier (i.e. BLAST plus HMM) is shown in the figure for comparison. The figure demonstrates: 1) The classifiers with refining technique outperform the one without refining technique, i.e. only with BLAST plus HMM; 2) The classifiers with balanced technique outperforms those without it. TransportTP with ensemble of balanced SVMs was chosen as our final strategy since it was more stable in performance than that of balanced random forest.

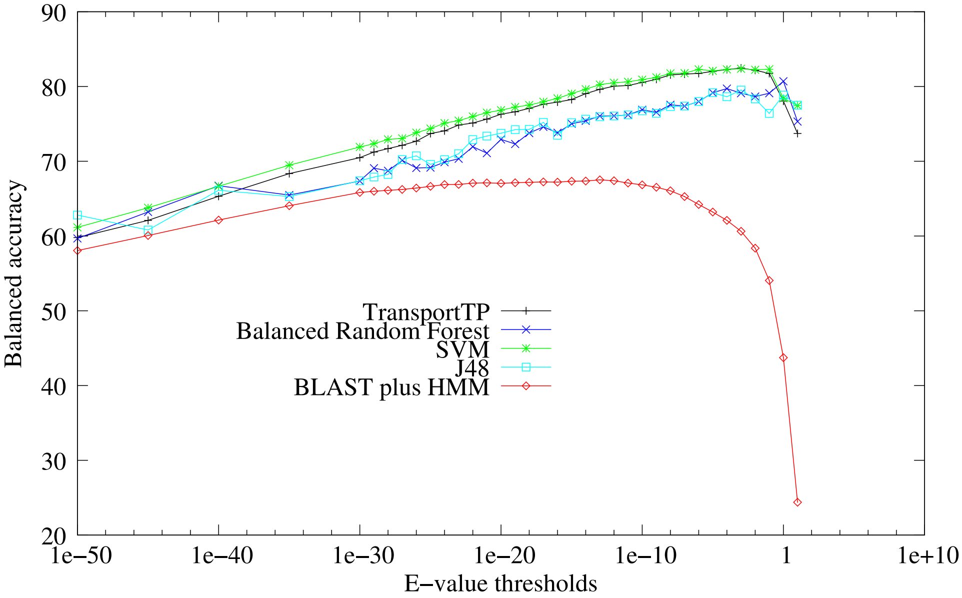

Figure 2: Balanced accuracy of TransportTP using different refining classifiers at e-value thresholds between 10 and 1e-50. The figure demonstrates: 1) TransportTP with refining classifiers outperforms that without it, i.e. only BLAST plus HMM. 2) TransportTP with refining classifiers is equivalent to that without it, i.e. only BLAST plus HMM, at most e-value thresholds. TransportTP with ensemble of balanced SVMs was chosen as the final strategy because it is more stable and performed equivalently in balanced accuracy and better precision with respect to the same recall, as shown in Figure 1.
