# Supplementary material for: TransportTP: A two-phase classification approach for membrane transporter prediction and characterization
Source: BMC Bioinformatics. 2009 Dec 14;10:418. doi: 10.1186/1471-2105-10-418 (PMC3087344; doi:10.1186/1471-2105-10-418)
Supplement: Additional file 7 — Mapping of Pfam domains to Transporter Classification (TC) families/superfamilies. This PDF table displays the mapping of Pfam domains to Transporter Classification families or superfamilies, using 1) cross-links in Pfam database, 2) automatic mapping between Pfam domains and TC families/superfamilies and 3) manual curation. [file 1471-2105-10-418-S7.PDF]

Pfam_ACC	TC_ACC	Pfam_DESCRIPTION	TC_DESCRIPTION	MAPPING APPROACH
PF00520	1.A.1	Ion channel	The Voltage-gated Ion Channel (VIC) Superfamily	Manual
PF07885	1.A.1	Ion channel	The Voltage-gated Ion Channel (VIC) Superfamily	Manual
PF00060	1.A.10	Ligand-gated ion channel	The Glutamate-gated Ion Channel (GIC) Family of Neurotransmitter Receptors	pfam db
PF00497	1.A.10	Bacterial extracellular solute-binding proteins, family 3	The Glutamate-gated Ion Channel (GIC) Family of Neurotransmitter Receptors	Auto
PF01094	1.A.10	Receptor family ligand binding region	The Glutamate-gated Ion Channel (GIC) Family of Neurotransmitter Receptors	Auto
PF10613	1.A.10	Ligated ion channel L-glutamate- and glycine-binding site	The Glutamate-gated Ion Channel (GIC) Family of Neurotransmitter Receptors	Auto
PF00654	1.A.11	Voltage gated chloride channel	The Ammonia Transporter Channel (Amt) Family	pfam db
PF00909	1.A.11	Ammonium Transporter Family	The Ammonia Transporter Channel (Amt) Family	Auto
PF00092	1.A.13	von Willebrand factor type A domain	The Epithelial Chloride Channel (E-ClC) Family	Manual
PF09315	1.A.13	Domain of unknown function (DUF1973)	The Epithelial Chloride Channel (E-ClC) Family	manual
PF03839	1.A.15	Translocation protein Sec62	The Non-selective Cation Channel-2 (NSCC2) Family	pfam db
PF00599	1.A.19	Influenza Matrix protein (M2)	The Type A Influenza Virus Matrix-2 Channel (M2-C) Family	pfam db
PF01007	1.A.2	Inward rectifier potassium channel	Inward Rectifier K+ Channel (IRK-C) Family	pfam db
PF08022	1.A.20	FAD-binding domain	The BCL2/Adenovirus E1B-interacting Protein 3 (BNip3) Family	manual
PF00452	1.A.21	Apoptosis regulator proteins, Bcl-2 family	The Bcl-2 (Bcl-2) Family	manual
PF02180	1.A.21	Bcl-2 homology region 4	The Bcl-2 (Bcl-2) Family	pfam db
PF01741	1.A.22	Large-conductance mechanosensitive channel, MscL	The Large Conductance Mechanosensitive Ion Channel (MscL) Family	pfam db
PF00924	1.A.23	Mechanosensitive ion channel	The Small Conductance Mechanosensitive Ion Channel (MscS) Family	pfam db
PF05552	1.A.23	Conserved TM helix	The Small Conductance Mechanosensitive Ion Channel (MscS) Family	pfam db
PF00029	1.A.24	Connexin	The Gap Junction-forming Connexin (Connexin) Family	pfam db
PF03508	1.A.24	Gap junction alpha-1 protein (Cx43)	The Gap Junction-forming Connexin (Connexin) Family	pfam db
PF10582	1.A.24	Gap junction channel protein cysteine-rich domain	The Gap Junction-forming Connexin (Connexin) Family	auto
PF00876	1.A.25	Innexin	The Gap Junction-forming Innexin (Innexin) Family	pfam db
PF02038	1.A.27	ATP1G1/PLM/MAT8 family	The Phospholemman (PLM) Family	pfam db
PF03253	1.A.28	Urea transporter	The Urea Transporter (UT) Family	pfam db
PF02293	1.A.29	AmiS/UreI family transporter	The Urea/Amide Channel (UAC) Family	pfam db
PF00520	1.A.3	Ion transport protein	The Ryanodine-Inositol 1,4,5-triphosphate Receptor Ca2+ Channel (RIR-CaC) Family	pfam db
PF00622	1.A.3	SPRY domain	The Ryanodine-Inositol 1,4,5-triphosphate Receptor Ca2+ Channel (RIR-CaC) Family	pfam db

PF01365	1.A.3	RIH domain	The Ryanodine-Inositol 1,4,5-triphosphate Receptor Ca2+ Channel (RIR-CaC) Family	pfam db
PF02026	1.A.3	RyR domain	The Ryanodine-Inositol 1,4,5-triphosphate Receptor Ca2+ Channel (RIR-CaC) Family	pfam db
PF02815	1.A.3	MIR domain	The Ryanodine-Inositol 1,4,5-triphosphate Receptor Ca2+ Channel (RIR-CaC) Family	pfam db
PF08454	1.A.3	RyR and IP3R Homology associated	The Ryanodine-Inositol 1,4,5-triphosphate Receptor Ca2+ Channel (RIR-CaC) Family	manual
PF08709	1.A.3	Inositol 1,4,5-trisphosphate/ryanodine receptor	The Ryanodine-Inositol 1,4,5-triphosphate Receptor Ca2+ Channel (RIR-CaC) Family	manual
PF00691	1.A.30	OmpA family	The H+- or Na+-translocating Bacterial Flagellar Motor 1ExbBD Outer Membrane Transport Energizer (Mot/Exb) Superfamily	auto
PF01618	1.A.30	MotA/TolQ/ExbB proton channel family	The H+- or Na+-translocating Bacterial Flagellar Motor 1ExbBD Outer Membrane Transport Energizer (Mot/Exb) Superfamily	pfam db
PF03544	1.A.30	Gram-negative bacterial tonB protein	The H+- or Na+-translocating Bacterial Flagellar Motor 1ExbBD Outer Membrane Transport Energizer (Mot/Exb) Superfamily	pfam db
PF00191	1.A.31	Annexin	The Annexin (Annexin) Family	pfam db
PF04159	1.A.32	NB glycoprotein	The Type B Influenza Virus NB Channel (NB-C) Family	pfam db
PF00012	1.A.33	Hsp70 protein	The Cation Channel-forming Heat Shock Protein-70 (Hsp70) Family	pfam db
PF00943	1.A.34	Alphavirus E2 glycoprotein	The Envelope Virus E1 Channel (EVE1-C) Family	pfam db
PF00944	1.A.34	Alphavirus core protein	The Envelope Virus E1 Channel (EVE1-C) Family	pfam db
PF01563	1.A.34	Alphavirus E3 glycoprotein	The Envelope Virus E1 Channel (EVE1-C) Family	pfam db
PF01589	1.A.34	Alphavirus E1 glycoprotein	The Envelope Virus E1 Channel (EVE1-C) Family	pfam db
PF01544	1.A.35	CorA-like Mg2+ transporter protein	The CorA Metal Ion Transporter (MIT) Family	pfam db
PF05934	1.A.36	Mid-1-related chloride channel (MCLC)	The Intracellular Chloride Channel (ICC) Family	pfam db
PF04103	1.A.37	CD20/IgE Fc receptor beta subunit family	The CD20 Ca2+ Channel (CD20) Family	pfam db
PF00073	1.A.38	picornavirus capsid protein	The Poliovirus 2B Viroporin (2B Viroporin) Family	manual
PF00947	1.A.38	Picornavirus core protein 2A	The Poliovirus 2B Viroporin (2B Viroporin) Family	manual
PF01552	1.A.38	Picornavirus 2B protein	The Poliovirus 2B Viroporin (2B Viroporin) Family	pfam db
PF02226	1.A.38	Picornavirus coat protein (VP4)	The Poliovirus 2B Viroporin (2B Viroporin) Family	manual
PF08727	1.A.38	Poliovirus 3A protein like	The Poliovirus 2B Viroporin (2B Viroporin) Family	manual
PF03021	1.A.39	Influenza C virus M2 protein	The Type C Influenza Virus CM2 Channel (CM2-C) Family	pfam db
PF03026	1.A.39	Influenza C virus M1 protein	The Type C Influenza Virus CM2 Channel (CM2-C) Family	manual
PF00023	1.A.4	Ankyrin repeat	The Transient Receptor Potential Ca2+ Channel (TRP-CC) Family	auto
PF00520	1.A.4	Ion transport protein	The Transient Receptor Potential Ca2+ Channel (TRP-CC) Family	pfam db

PF00558	1.A.40	Vpu protein	The Human Immunodeficiency Virus Type I, HIV-1 (Retrovirdiac) Vpu Channel (Vpu-C) Family	pfam db
PF07204	1.A.41	Orthoreovirus membrane fusion protein p10	The Avian Reovirus p10 Viroporin (p10) Family	manual
PF00522	1.A.42	VPR/VPX protein	The HIV Viral Protein R (Vpr) Family	pfam db
PF01062	1.A.46	Bestrophin	The Anion Channel-forming Bestrophin (Bestrophin) Family	manual
PF04906	1.A.48	Tweety	The Anion Channel Tweety (Tweety) Family	manual
PF00520	1.A.5	Ion transport protein	The Polycystin Cation Channel (PCC) Family	auto
PF00801	1.A.5	PKD domain	The Polycystin Cation Channel (PCC) Family	auto
PF08016	1.A.5	Polycystin cation channel	The Polycystin Cation Channel (PCC) Family	auto
PF04272	1.A.50	Phospholamban	The Phospholamban (Ca2+-channel and Ca2+-ATPase Regulator) (PLB) Family	manual
PF04145	1.A.56	Ctr copper transporter family	The Copper Transporter (Ctr) Family	auto
PF00858	1.A.6	Amiloride-sensitive sodium channel	The Epithelial Na+ Channel (ENaC) Family	pfam db
PF00864	1.A.7	ATP P2X receptor	ATP-gated Cation Channel (ACC) Family	pfam db
PF00230	1.A.8	Major intrinsic protein	The Major Intrinsic Protein (MIP) Family	pfam db
PF02931	1.A.9	Neurotransmitter-gated ion-channel ligand binding domain	The Neurotransmitter Receptor, Cys loop, Ligand-gated Ion Channel (LIC) Family	pfam db
PF02932	1.A.9	Neurotransmitter-gated ion-channel transmembrane region	The Neurotransmitter Receptor, Cys loop, Ligand-gated Ion Channel (LIC) Family	pfam db
PF00267	1.B.1	Gram-negative porin	The General Bacterial Porin (GBP) Family	auto
PF03502	1.B.10	Nucleoside-specific channel-forming protein, Tsx	The Nucleoside-specific Channel-forming Outer Membrane Porin (Tsx) Family	pfam db
PF00577	1.B.11	Fimbrial Usher protein	The Outer Membrane Fimbrial Usher Porin (FUP) Family	pfam db
PF03797	1.B.12	Autotransporter beta-domain	The Autotransporter-1 (AT-1) Family	pfam db
PF00593	1.B.14	TonB dependent receptor	The Outer Membrane Receptor (OMR) Family	pfam db
PF07715	1.B.14	TonB-dependent Receptor Plug Domain	The Outer Membrane Receptor (OMR) Family	auto
PF02321	1.B.17	Outer membrane efflux protein	The Outer Membrane Factor (OMF) Family	pfam db
PF02563	1.B.18	Polysaccharide biosynthesis/export protein	The Outer Membrane Auxiliary (OMA) Protein Family	pfam db
PF10531	1.B.18	SLBB domain	The Outer Membrane Auxiliary (OMA) Protein Family	auto
PF04966	1.B.19	Carbohydrate-selective porin, OprB family	The Glucose-selective OprB Porin (OprB) Family	pfam db
PF01308	1.B.2	Chlamydia major outer membrane protein	The Chlamydial Porin (CP) Family	pfam db
PF08479	1.B.20	POTRA domain, ShlB-type	The Two-Partner Secretion (TPS) Family	manual
PF06178	1.B.21	Oligogalacturonate-specific porin protein (KdgM)	1.B.21 The OmpG Porin (OmpG) Family	pfam db
PF00263	1.B.22	Bacterial type II and III secretion system protein	The Outer Bacterial Membrane Secretin (Secretin) Family	pfam db
PF03958	1.B.22	Bacterial type II/III secretion system short domain	The Outer Bacterial Membrane Secretin (Secretin) Family	pfam db
PF09203	1.B.24	MspA	The Mycobacterial Porin (MBP) Family	manual
PF03573	1.B.25	outer membrane porin, OprD family	The Outer Membrane Porin (OPr) Family	pfam db

PF01856	1.B.27	Helicobacter outer membrane protein	The Helicobacter Outer Membrane Porin (HOP) Family	pfam db
PF02264	1.B.3	LamB porin	The Sugar Porin (SP) Family	pfam db
PF05538	1.B.31	Campylobacter major outer membrane protein	The Campylobacter jejuni Major Outer Membrane Porin (MomP) Family	pfam db
PF07244	1.B.33	Surface antigen variable number repeat	The Outer Membrane Protein Insertion Porin (OmpIP) Family	auto
PF06178	1.B.35	Oligogalacturonate-specific porin protein (KdgM)	The Oligogalacturonate-specific Porin (KdgM) Family	pfam db
PF05628	1.B.36	Borrelia membrane protein P13	The Borrelia Porin p13 (BP-p13) Family	pfam db
PF02707	1.B.38	Major Outer Sheath Protein N-terminal region	The Treponema Porin Major Surface Protein (TP-MSP) Family	pfam db
PF02722	1.B.38	Major Outer Sheath Protein C-terminal region	The Treponema Porin Major Surface Protein (TP-MSP) Family	pfam db
PF03922	1.B.39	OmpW family	The Bacterial Porin, OmpW (OmpW) Family	pfam db
PF02530	1.B.4	Porin subfamily	The Brucella-Rhizobium Porin (BRP) Family	pfam db
PF05244	1.B.4	Brucella outer membrane protein 2	The Brucella-Rhizobium Porin (BRP) Family	pfam db
PF03895	1.B.40	YadA-like C-terminal region	The Autotransporter-2 (AT-2) Family	auto
PF05658	1.B.40	Hep_Hag	The Autotransporter-2 (AT-2) Family	auto
PF05662	1.B.40	Haemagglutinin	The Autotransporter-2 (AT-2) Family	auto
PF07396	1.B.5	Phosphate-selective porin O and P	The Pseudomonas OprP Porin (POP) Family	manual
PF00691	1.B.6	OmpA family	The OmpA-OmpF Porin (OOP) Family	pfam db
PF01389	1.B.6	OmpA-like transmembrane domain	The OmpA-OmpF Porin (OOP) Family	pfam db
PF05736	1.B.6	OprF membrane domain	The OmpA-OmpF Porin (OOP) Family	pfam db
PF01459	1.B.8	Eukaryotic porin	The Mitochondrial and Plastid Porin (MPP) Family	pfam db
PF03349	1.B.9	Outer membrane protein transport protein (OMPP1/FadL/TodX)	The FadL Outer Membrane Protein (FadL) Family	pfam db
PF01024	1.C.1	Colicin pore forming domain	The Channel-forming Colicin (Colicin) Family	pfam db
PF06109	1.C.10	Haemolysin E (HlyE)	The Pore-forming Haemolysin E (HlyE) Family	pfam db
PF02382	1.C.11	RTX N-terminal domain	The Pore-forming RTX Toxin (RTX-toxin) Family	pfam db
PF01289	1.C.12	Thiol-activated cytolysin	The Cholesterol-binding, Thiol-activated Cytolysin (TAC) Family	pfam db
PF01117	1.C.14	Aerolysin toxin	The Cytohemolysin (CHL) Family	pfam db
PF00095	1.C.15	WAP-type (Whey Acidic Protein) 'four-disulfide core'	The Whipworm Stichosome Porin (WSP) Family	pfam db
PF00272	1.C.17	Cecropin family	The Cecropin (Cecropin) Family	pfam db
PF01372	1.C.18	Melittin	The Melittin (Melittin) Family	pfam db
PF00323	1.C.19	Mammalian defensin	The Defensin (Defensin) Family	pfam db
PF00879	1.C.19	Defensin propeptide	The Defensin (Defensin) Family	pfam db
PF00555	1.C.2	delta endotoxin	The Channel-forming delta-Endotoxin Insecticidal Crystal Protein (ICP) Family	pfam db
PF03944	1.C.2	delta endotoxin	The Channel-forming delta-Endotoxin Insecticidal Crystal Protein (ICP) Family	pfam db
PF03945	1.C.2	delta endotoxin, N-terminal domain	The Channel-forming delta-Endotoxin Insecticidal Crystal Protein (ICP) Family	pfam db
PF02052	1.C.20	Gallidermin	The Nisin (Nisin) Family	auto

PF04604	1.C.21	Type-A lantibiotic	The Lacticin 481 (Lacticin 481) Family	pfam db
PF04369	1.C.22	Lactococcin-like family	The Lactococcin A (Lactococcin A) Family	pfam db
PF01721	1.C.24	Class II bacteriocin	The Pediocin (Pediocin) Family	pfam db
PF09221	1.C.28	Bacteriocin AS-48	The Bacteriocin AS-48 Cyclic Polypeptide (Bacteriocin AS-48) Family	manual
PF01117	1.C.3	Aerolysin toxin	The alpha-Hemolysin Channel-forming Toxin (alphaHL) Family	pfam db
PF07968	1.C.3	Leukocidin/Hemolysin toxin family	The alpha-Hemolysin Channel-forming Toxin (alphaHL) Family	auto
PF00666	1.C.33	Cathelicidin	The Cathelicidin (Cathelicidin) Family	pfam db
PF03489	1.C.35	Saposin-like type B, region 2	The Amoebapore (Amoebapore) Family	auto
PF03518	1.C.36	Salmonella/Shigella invasin protein B	The Bacterial Type III-Target Cell Pore (IIITCP) Family	pfam db
PF04888	1.C.36	Secretion system effector C (SseC) like family	The Bacterial Type III-Target Cell Pore (IIITCP) Family	pfam db
PF06369	1.C.38	Sea anemone cytotoxic protein	The Pore-forming Equinatoxin (Equinatoxin) Family	pfam db
PF01823	1.C.39	MAC/Perforin domain	The Complement Protein C9 (CPC9) Family	pfam db
PF01117	1.C.4	Aerolysin toxin	The Aerolysin Channel-forming Toxin (Aerolysin) Family	pfam db
PF01273	1.C.40	LBP / BPI / CETP family, N-terminal domain	The Bactericidal Permeability Increasing Protein (BPIP) Family	pfam db
PF02886	1.C.40	LBP / BPI / CETP family, C-terminal domain	The Bactericidal Permeability Increasing Protein (BPIP) Family	pfam db
PF05791	1.C.41	Bacillus haemolytic enterotoxin (HBL)	The Tripartite Haemolysin BL (HBL) Family	pfam db
PF03495	1.C.42	Clostridial binary toxin B/anthrax toxin PA	The Channel-forming Bacillus anthrax Protective Antigen (BAPA) Family	pfam db
PF07691	1.C.42	PA14 domain	The Channel-forming Bacillus anthrax Protective Antigen (BAPA) Family	manual
PF00321	1.C.44	Plant thionin	The Plant Thionine (PT) Family	pfam db
PF00304	1.C.45	Gamma-thionin family	The Plant Defensin (PD) Family	manual
PF00212	1.C.46	Atrial natriuretic peptide	The C-type Natriuretic Peptide (CNP) Family	pfam db
PF01097	1.C.47	Arthropod defensin	The Insect Defensin (Insect Defensin) Family	pfam db
PF00377	1.C.48	Prion/Doppel alpha-helical domain	The Prion Peptide Fragment (PrP-F) Family	pfam db
PF03991	1.C.48	Copper binding octapeptide repeat	The Prion Peptide Fragment (PrP-F) Family	pfam db
PF00214	1.C.49	Calcitonin / CGRP / IAPP family	The Cytotoxic Amylin (Amylin) Family	pfam db
PF03318	1.C.5	Clostridium epsilon toxin ETX/Bacillus mosquitocidal toxin MTX2	The Channel-forming epsilon-toxin (epsilon-toxin) Family	pfam db
PF02177	1.C.50	Amyloid A4 extracellular domain	The Amyloid beta-Protein Peptide (AbetaPP) Family	pfam db
PF03494	1.C.50	Beta-amyloid peptide (beta-APP)	The Amyloid beta-Protein Peptide (AbetaPP) Family	manual
PF03032	1.C.52	Brevenin/esculentin/gaegurin/rugosin family	The Dermaseptin (Dermaseptin) Family	pfam db
PF02258	1.C.54	Shiga-like toxin beta subunit	The Shiga Toxin B-Chain (ST-B) Family	pfam db
PF07229	1.C.55	VirE2	The Agrobacterial VirE2 Target Host Cell Membrane Anion Channel (VirE2) Family	manual
PF04877	1.C.56	HrpZ	The Pseudomanas syringae HrpZ Target Host Cell Membrane Cation Channel (HrpZ) Family	pfam db

PF03505	1.C.59	Clostridium enterotoxin	The Clostridium perfringens Enterotoxin (CPE) Family	pfam db
PF08107	1.C.62	Pleurocidin family	The Pseudopleuronectes americanus (flounder) Pleurocidin (Pleurocidin) Family	manual
PF05819	1.C.65	NolX protein	The Type III Secretion System Plant Host Cell Membrane Pore-forming HrpF (HrpF) Family	pfam db
PF08025	1.C.68	Spider antimicrobial peptide	The Channel-forming Oxyopinin Peptide (Oxyopinin) Family	manual
PF01324	1.C.7	Diphtheria toxin, R domain	The Diphtheria Toxin (DT) Family	pfam db
PF02763	1.C.7	Diphtheria toxin, C domain	The Diphtheria Toxin (DT) Family	pfam db
PF02764	1.C.7	Diphtheria toxin, T domain	The Diphtheria Toxin (DT) Family	pfam db
PF07373	1.C.70	CAMP factor (Cfa)	The Streptococcal Pore-forming CAMP Factor (CAMP-F) Family	manual
PF09009	1.C.73	Exotoxin A catalytic	The Pseudomonas Exotoxin A (P-ExoA) Family	manual
PF09101	1.C.73	Exotoxin A binding	The Pseudomonas Exotoxin A (P-ExoA) Family	manual
PF09102	1.C.73	Exotoxin A, targeting	The Pseudomonas Exotoxin A (P-ExoA) Family	manual
PF01742	1.C.8	Clostridial neurotoxin zinc protease	The Botulinum and Tetanus Toxin (BTT) Family	manual
PF07951	1.C.8	Clostridium neurotoxin, C-terminal receptor binding	The Botulinum and Tetanus Toxin (BTT) Family	manual
PF07952	1.C.8	Clostridium neurotoxin, Translocation domain	The Botulinum and Tetanus Toxin (BTT) Family	manual
PF07953	1.C.8	Clostridium neurotoxin, N-terminal receptor binding	The Botulinum and Tetanus Toxin (BTT) Family	manual
PF02691	1.C.9	Vacuolating cyotoxin	The Vacuolating Cytotoxin (VacA) Family	pfam db
PF04971	1.E.1	Lysis protein S	The P21 Holin S (P21 Holin) Family	pfam db
PF05105	1.E.10	Holin family	The Bacillus subtilis phi29 Holin (phi29 Holin) Family	pfam db
PF04531	1.E.11	Bacteriophage holin	The phi11 Holin (phi11 Holin) Family	pfam db
PF03788	1.E.14	LrgA family	The LrgA Holin (LrgA Holin) Family	pfam db
PF05105	1.E.16	Holin family	The Cph1 Holin (Cph1 Holin) Family	pfam db
PF05102	1.E.17	holin, BlyA family	The BlyA Holin (BlyA Holin) Family	pfam db
PF05105	1.E.19	Holin family	The Clostridium difficile TcdE Holin (TcdE Holin) Family	pfam db
PF05106	1.E.2	Phage holin family (Lysis protein S)	The lambda Holin S (lambda Holin) Family	pfam db
PF06946	1.E.21	Phage holin	The Listeria Phage A118 Holin (hol118) Family	manual
PF04550	1.E.3	Phage holin family 2	The P2 Holin TM (P2 Holin) Family	pfam db
PF00822	1.G.1	PMP-22/EMP/MP20/Claudin family	The Claudin Tight Junction (Claudin) Family	auto
PF00083	2.A.1	Sugar (and other) transporter	The Major Facilitator Superfamily (MFS)	pfam db
PF01306	2.A.1	LacY proton/sugar symporter	The Major Facilitator Superfamily (MFS)	pfam db
PF03825	2.A.1	Nucleoside H+ symporter	The Major Facilitator Superfamily (MFS)	pfam db
PF05977	2.A.1	Bacterial protein of unknown function (DUF894)	The Major Facilitator Superfamily (MFS)	pfam db
PF07672	2.A.1	Mycoplasma MFS transporter	The Major Facilitator Superfamily (MFS)	pfam db
PF07690	2.A.1	Major Facilitator Superfamily	The Major Facilitator Superfamily (MFS)	manual
PF03812	2.A.10	2-keto-3-deoxygluconate permease	The 2-Keto-3-Deoxygluconate Transporter (KDGT) Family	pfam db
PF03600	2.A.11	Citrate transporter	The Citrate-Mg2+:H+ (CitM) Citrate-	pfam db

			Ca2+:H+ (CitH) Symporter (CitMHS) Family	
PF03219	2.A.12	TLC ATP/ADP transporter	The ATP:ADP Antiporter (AAA) Family	pfam db
PF03605	2.A.13	Anaerobic c4-dicarboxylate membrane transporter	The C4-Dicarboxylate Uptake (Dcu) Family	pfam db
PF02652	2.A.14	L-lactate permease	The Lactate Permease (LctP) Family	pfam db
PF02028	2.A.15	BCCT family transporter	The Betaine/Carnitine/Choline Transporter (BCCT) Family	pfam db
PF03595	2.A.16	C4-dicarboxylate transporter/malic acid transport protein	The Telurite-resistance/Dicarboxylate Transporter (TDT) Family	pfam db
PF00854	2.A.17	POT family	The Proton-dependent Oligopeptide Transporter (POT) Family	pfam db
PF01490	2.A.18	Transmembrane amino acid transporter protein	The Amino Acid/Auxin Permease (AAAP) Family	pfam db
PF01699	2.A.19	Sodium/calcium exchanger protein	The Ca2+:Cation Antiporter (CaCA) Family	pfam db
PF00083	2.A.2	Sugar (and other) transporter	The Glycoside-Pentoside-Hexuronide (GPH):Cation Symporter Family	pfam db
PF07690	2.A.2	Major Facilitator Superfamily	The Glycoside-Pentoside-Hexuronide (GPH):Cation Symporter Family	auto
PF01384	2.A.20	Phosphate transporter family	The Inorganic Phosphate Transporter (PiT) Family	pfam db
PF00474	2.A.21	Sodium:solute symporter family	The Solute:Sodium Symporter (SSS) Family	pfam db
PF00209	2.A.22	Sodium:neurotransmitter symporter family	The Neurotransmitter:Sodium Symporter (NSS) Family	manual
PF00375	2.A.23	Sodium:dicarboxylate symporter family	The Dicarboxylate/Amino Acid:Cation (Na+ or H+) Symporter (DAACS) Family	pfam db
PF03390	2.A.24	2-hydroxycarboxylate transporter family	The 2-Hydroxycarboxylate Transporter (2-HCT) Family	pfam db
PF01235	2.A.25	Sodium:alanine symporter family	The Alanine or Glycine:Cation Symporter (AGCS) Family	pfam db
PF05525	2.A.26	Branched-chain amino acid transport protein	The Branched Chain Amino Acid:Cation Symporter (LIVCS) Family	pfam db
PF03616	2.A.27	Sodium/glutamate symporter	The Glutamate:Na+ Symporter (ESS) Family	pfam db
PF01758	2.A.28	Sodium Bile acid symporter family	The Bile Acid:Na+ Symporter (BASS) Family	pfam db
PF00153	2.A.29	Mitochondrial carrier protein	The Mitochondrial Carrier (MC) Family	pfam db
PF04082	2.A.29	Fungal specific transcription factor domain	The Mitochondrial Carrier (MC) Family	pfam db
PF00324	2.A.3	Amino acid permease	The Amino Acid-Polyamine-Organocation (APC) Family	pfam db
PF03845	2.A.3	Spore germination protein	The Amino Acid-Polyamine-Organocation (APC) Family	pfam db
PF00324	2.A.30	Amino acid permease	The Cation-Chloride Cotransporter (CCC) Family	auto
PF03522	2.A.30	K-Cl Co-transporter type 1 (KCC1)	The Cation-Chloride Cotransporter (CCC) Family	pfam db
PF08403	2.A.30	Amino acid permease N-terminal	The Cation-Chloride Cotransporter (CCC) Family	auto
PF00955	2.A.31	HCO3- transporter family	The Anion Exchanger (AE) Family	pfam db
PF07565	2.A.31	Band 3 cytoplasmic domain	The Anion Exchanger (AE) Family	pfam db
PF03842	2.A.32	Silicon transporter	The Silicon Transporter (Sit) Family	pfam db
PF06965	2.A.33	Na+/H+ antiporter 1	The NhaA Na+:H+ Antiporter (NhaA)	manual

			Family	
PF06450	2.A.34	Bacterial Na+/H+ antiporter B (NhaB)	The NhaB Na+:H+ Antiporter (NhaB) Family	manual
PF03553	2.A.35	Na+/H+ antiporter family	The NhaC Na+:H+ Antiporter (NhaC) Family	pfam db
PF00999	2.A.36	Sodium/hydrogen exchanger family	The Monovalent Cation:Proton Antiporter-1 (CPA1) Family	pfam db
PF00999	2.A.37	Sodium/hydrogen exchanger family	The Monovalent Cation:Proton Antiporter-2 (CPA2) Family	pfam db
PF02386	2.A.38	Cation transport protein	The K+ Transporter (Trk) Family	pfam db
PF02133	2.A.39	Permease for cytosine/purines, uracil, thiamine, allantoin	The Nucleobase:Cation Symporter-1 (NCS1) Family	pfam db
PF01545	2.A.4	Cation efflux family	The Cation Diffusion Facilitator (CDF) Family	pfam db
PF00860	2.A.40	Permease family	The Nucleobase:Cation Symporter-2 (NCS2) Family	pfam db
PF01773	2.A.41	Na+ dependent nucleoside transporter N-terminus	The Concentrative Nucleoside Transporter (CNT) Family	pfam db
PF07662	2.A.41	Na+ dependent nucleoside transporter C-terminus	The Concentrative Nucleoside Transporter (CNT) Family	pfam db
PF07670	2.A.41	Nucleoside recognition	The Concentrative Nucleoside Transporter (CNT) Family	auto
PF03222	2.A.42	Tryptophan/tyrosine permease family	The Hydroxy/Aromatic Amino Acid Permease (HAAAP) Family	pfam db
PF04193	2.A.43	PQ loop repeat	The Lysosomal Cystine Transporter (LCT) Family	manual
PF01226	2.A.44	Formate/nitrite transporter	The Formate-Nitrite Transporter (FNT) Family	pfam db
PF02040	2.A.45	Arsenical pump membrane protein	The Arsenite-Antimonite (ArsB) Efflux Family	pfam db
PF03594	2.A.46	Benzoate membrane transport protein	The Benzoate:H+ Symporter (BenE) Family	pfam db
PF00939	2.A.47	Sodium:sulfate symporter transmembrane region	The Divalent Anion:Na+ Symporter (DASS) Family	pfam db
PF01770	2.A.48	Reduced folate carrier	The Reduced Folate Carrier (RFC) Family	pfam db
PF00571	2.A.49	CBS domain pair	The Chloride Carrier/Channel (ClC) Family	auto
PF00654	2.A.49	Voltage gated chloride channel	The Chloride Carrier/Channel (ClC) Family	auto
PF00909	2.A.49	Ammonium Transporter Family	The Chloride Carrier/Channel (ClC) Family	pfam db
PF02535	2.A.5	ZIP Zinc transporter	The Zinc (Zn2+)-Iron (Fe2+) Permease (ZIP) Family	pfam db
PF03062	2.A.50	MBOAT family	The Glycerol Uptake (GUP) Family	manual
PF02417	2.A.51	Chromate transporter	The Chromate Ion Transporter (CHR) Family	pfam db
PF03824	2.A.52	High-affinity nickel-transport protein	The Ni2+-Co2+ Transporter (NiCoT) Family	pfam db
PF00916	2.A.53	Sulfate transporter family	The Sulfate Permease (SulP) Family	pfam db
PF01740	2.A.53	STAS domain	The Sulfate Permease (SulP) Family	manual
PF03820	2.A.54	Tricarboxylate carrier	The Mitochondrial Tricarboxylate Carrier (MTC) Family	pfam db
PF01566	2.A.55	Natural resistance-associated macrophage protein	The Metal Ion (Mn2+-iron) Transporter (Nramp) Family	pfam db
PF03480	2.A.56	Bacterial extracellular solute-binding protein, family 7	The Tripartite ATP-independent Periplasmic Transporter (TRAP-T) Family	pfam db
PF04290	2.A.56	Tripartite ATP-independent periplasmic transporters, DctQ component	The Tripartite ATP-independent Periplasmic Transporter (TRAP-T) Family	pfam db

PF01733	2.A.57	Nucleoside transporter	The Equilibrative Nucleoside Transporter (ENT) Family	pfam db
PF02690	2.A.58	Na+/Pi-cotransporter	The Phosphate:Na+ Symporter (PNaS) Family	pfam db
PF01758	2.A.59	Sodium Bile acid symporter family	The Arsenical Resistance-3 (ACR3) Family	pfam db
PF00529	2.A.6	HlyD family secretion protein	The Resistance-Nodulation-Cell Division (RND) Superfamily	pfam db
PF00873	2.A.6	AcrB/AcrD/AcrF family	The Resistance-Nodulation-Cell Division (RND) Superfamily	pfam db
PF02321	2.A.6	Outer membrane efflux protein	The Resistance-Nodulation-Cell Division (RND) Superfamily	pfam db
PF03137	2.A.60	Organic Anion Transporter Polypeptide (OATP) family	The Organo Anion Transporter (OAT) Family	pfam db
PF07648	2.A.60	Kazal-type serine protease inhibitor domain	The Organo Anion Transporter (OAT) Family	auto
PF07690	2.A.60	Major Facilitator Superfamily	The Organo Anion Transporter (OAT) Family	auto
PF03606	2.A.61	C4-dicarboxylate anaerobic carrier	The C4-dicarboxylate Uptake C (DcuC) Family	pfam db
PF01899	2.A.63	Na+/H+ ion antiporter subunit	The Monovalent Cation (K+ or Na+):Proton Antiporter-3 (CPA3) Family	pfam db
PF03334	2.A.63	Na+/H+ antiporter subunit	The Monovalent Cation (K+ or Na+):Proton Antiporter-3 (CPA3) Family	pfam db
PF04039	2.A.63	Domain related to MnhB subunit of Na+/H+ antiporter	The Monovalent Cation (K+ or Na+):Proton Antiporter-3 (CPA3) Family	pfam db
PF04066	2.A.63	Multiple resistance and pH regulation protein F (MrpF / PhaF)	The Monovalent Cation (K+ or Na+):Proton Antiporter-3 (CPA3) Family	pfam db
PF00902	2.A.64	Sec-independent protein translocase protein (TatC)	The Twin Arginine Targeting (Tat) Family	pfam db
PF02416	2.A.64	mttA/Hcf106 family	The Twin Arginine Targeting (Tat) Family	pfam db
PF01554	2.A.66	MatE	The Multidrug/Oligosaccharidyl-lipid/Polysaccharide (MOP) Flippase Superfamily	pfam db
PF03023	2.A.66	MviN-like protein	The Multidrug/Oligosaccharidyl-lipid/Polysaccharide (MOP) Flippase Superfamily	pfam db
PF03169	2.A.67	OPT oligopeptide transporter protein	The Oligopeptide Transporter (OPT) Family	pfam db
PF03806	2.A.68	AbgT putative transporter family	The p-Aminobenzoyl-glutamate Transporter (AbgT) Family	pfam db
PF03547	2.A.69	Membrane transport protein	The Auxin Efflux Carrier (AEC) Family	pfam db
PF00892	2.A.7	Integral membrane protein DUF6	The Drug/Metabolite Transporter (DMT) Superfamily	pfam db
PF00893	2.A.7	Small Multidrug Resistance protein	The Drug/Metabolite Transporter (DMT) Superfamily	pfam db
PF03151	2.A.7	Triose-phosphate Transporter family	The Drug/Metabolite Transporter (DMT) Superfamily	pfam db
PF04142	2.A.7	Nucleotide-sugar transporter	The Drug/Metabolite Transporter (DMT) Superfamily	pfam db
PF06379	2.A.7	L-rhamnose-proton symport protein (RhaT)	The Drug/Metabolite Transporter (DMT) Superfamily	pfam db
PF07857	2.A.7	CEO family (DUF1632)	The Drug/Metabolite Transporter (DMT) Superfamily	pfam db
PF08449	2.A.7	UAA transporter family	The Drug/Metabolite Transporter (DMT) Superfamily	pfam db

PF03817	2.A.70	Malonate transporter MadL subunit	The Malonate:Na+ Symporter (MSS) Family	pfam db
PF03818	2.A.70	Malonate/sodium symporter MadM subunit	The Malonate:Na+ Symporter (MSS) Family	pfam db
PF03092	2.A.71	BT1 family	The Folate-Biopterin Transporter (FBT) Family	pfam db
PF02705	2.A.72	K+ potassium transporter	The K+ Uptake Permease (KUP) Family	pfam db
PF02667	2.A.73	Short chain fatty acid transporter	The Short Chain Fatty Acid Uptake (AtoE) Family	manual
PF03821	2.A.74	Golgi 4-transmembrane spanning transporter	The 4 TMS Multidrug Endosomal Transporter (MET) Family	pfam db
PF01810	2.A.75	LysE type translocator	The L-Lysine Exporter (LysE) Family	pfam db
PF01810	2.A.76	LysE type translocator	The Resistance to Homoserine/Threonine (RhtB) Family	auto
PF03596	2.A.77	Cadmium resistance transporter	The Cadmium Resistance (CadD) Family	pfam db
PF03591	2.A.78	AzlC protein	The Branched Chain Amino Acid Exporter (LIV-E) Family	pfam db
PF05437	2.A.78	Branched-chain amino acid transport protein (AzlD)	The Branched Chain Amino Acid Exporter (LIV-E) Family	pfam db
PF02447	2.A.8	GntP family permease	The Gluconate:H+ Symporter (GntP) Family	pfam db
PF01970	2.A.80	Integral membrane protein DUF112	The Tricarboxylate Transporter (TTT) Family	pfam db
PF03401	2.A.80	Bordetella uptake gene (bug) product	The Tricarboxylate Transporter (TTT) Family	pfam db
PF06826	2.A.81	Predicted Permease Membrane Region	The Aspartate:Alanine Exchanger (AAE) Family	manual
PF02096	2.A.9	60Kd inner membrane protein	The Cytochrome Oxidase Biogenesis (Oxa1) Family	pfam db
PF01618	2.C.1	MotA/TolQ/ExbB proton channel family	The TonB-ExbB-ExbD/TolA-TolQ-TolR (TonB) Family of Auxiliary Proteins for Energization of Outer Membrane Receptor (OMR)-mediated Active Transport	pfam db
PF02472	2.C.1	Biopolymer transport protein ExbD/TolR	The TonB-ExbB-ExbD/TolA-TolQ-TolR (TonB) Family of Auxiliary Proteins for Energization of Outer Membrane Receptor (OMR)-mediated Active Transport	pfam db
PF03544	2.C.1	Gram-negative bacterial tonB protein	The TonB-ExbB-ExbD/TolA-TolQ-TolR (TonB) Family of Auxiliary Proteins for Energization of Outer Membrane Receptor (OMR)-mediated Active Transport	pfam db
PF00005	3.A.1	ABC transporter	The ATP-binding Cassette (ABC) Superfamily	pfam db
PF00496	3.A.1	Bacterial extracellular solute-binding proteins, family 5 Middle	The ATP-binding Cassette (ABC) Superfamily	pfam db
PF00497	3.A.1	Bacterial extracellular solute-binding proteins, family 3	The ATP-binding Cassette (ABC) Superfamily	pfam db
PF00528	3.A.1	Binding-protein-dependent transport system inner membrane component	The ATP-binding Cassette (ABC) Superfamily	pfam db
PF00529	3.A.1	HlyD family secretion protein	The ATP-binding Cassette (ABC) Superfamily	pfam db
PF00532	3.A.1	Periplasmic binding proteins and sugar binding domain of the LacI family	The ATP-binding Cassette (ABC) Superfamily	pfam db
PF00664	3.A.1	ABC transporter transmembrane region	The ATP-binding Cassette (ABC) Superfamily	pfam db

PF01032	3.A.1	FecCD transport family	The ATP-binding Cassette (ABC) Superfamily	pfam db
PF01061	3.A.1	ABC-2 type transporter	The ATP-binding Cassette (ABC) Superfamily	pfam db
PF01094	3.A.1	Receptor family ligand binding region	The ATP-binding Cassette (ABC) Superfamily	pfam db
PF01297	3.A.1	Periplasmic solute binding protein family	The ATP-binding Cassette (ABC) Superfamily	pfam db
PF01497	3.A.1	Periplasmic binding protein	The ATP-binding Cassette (ABC) Superfamily	pfam db
PF01547	3.A.1	Bacterial extracellular solute-binding protein	The ATP-binding Cassette (ABC) Superfamily	pfam db
PF01578	3.A.1	Cytochrome C assembly protein	The ATP-binding Cassette (ABC) Superfamily	pfam db
PF01891	3.A.1	Cobalt uptake substrate-specific transmembrane region	The ATP-binding Cassette (ABC) Superfamily	pfam db
PF02361	3.A.1	Cobalt transport protein	The ATP-binding Cassette (ABC) Superfamily	pfam db
PF02553	3.A.1	Cobalt transport protein component CbiN	The ATP-binding Cassette (ABC) Superfamily	pfam db
PF02653	3.A.1	Branched-chain amino acid transport system / permease component	The ATP-binding Cassette (ABC) Superfamily	pfam db
PF03379	3.A.1	CcmB protein	The ATP-binding Cassette (ABC) Superfamily	pfam db
PF04069	3.A.1	Substrate binding domain of ABC-type glycine betaine transport system	The ATP-binding Cassette (ABC) Superfamily	pfam db
PF05025	3.A.1	RbsD / FucU transport protein family	The ATP-binding Cassette (ABC) Superfamily	pfam db
PF10670	3.A.1	Nickel uptake substrate-specific transmembrane region	The ATP-binding Cassette (ABC) Superfamily	pfam db
PF03030	3.A.10	Inorganic H+ pyrophosphatase	The H+-translocating Pyrophosphatase (H+-PPase) Family	pfam db
PF03772	3.A.11	Competence protein	The Bacterial Competence-related DNA Transformation Transporter (DNA-T) Family	pfam db
PF01580	3.A.12	FtsK/SpoIIIE family	The Septal DNA Translocator (S-DNA-T) Family	auto
PF05707	3.A.13	Zonular occludens toxin (Zot)	The Filamentous Phage Exporter (FPhE) Family	pfam db
PF00437	3.A.14	Type II/IV secretion system protein	The Fimbrilin/Protein Exporter (FPE) Family	pfam db
PF00482	3.A.14	Bacterial type II secretion system protein F domain	The Fimbrilin/Protein Exporter (FPE) Family	pfam db
PF00263	3.A.15	Bacterial type II and III secretion system protein	The Outer Membrane Protein Secreting Main Terminal Branch (MTB) Family	pfam db
PF00437	3.A.15	Type II/IV secretion system protein	The Outer Membrane Protein Secreting Main Terminal Branch (MTB) Family	pfam db
PF00482	3.A.15	Bacterial type II secretion system protein F domain	The Outer Membrane Protein Secreting Main Terminal Branch (MTB) Family	pfam db
PF01203	3.A.15	Bacterial type II secretion system protein N	The Outer Membrane Protein Secreting Main Terminal Branch (MTB) Family	pfam db
PF02501	3.A.15	Bacterial type II secretion system protein I/J	The Outer Membrane Protein Secreting Main Terminal Branch (MTB) Family	pfam db
PF03934	3.A.15	General secretion pathway protein K	The Outer Membrane Protein Secreting Main Terminal Branch (MTB) Family	pfam db

PF03958	3.A.15	Bacterial type II/III secretion system short domain	The Outer Membrane Protein Secreting Main Terminal Branch (MTB) Family	pfam db
PF04612	3.A.15	General secretion pathway, M protein	The Outer Membrane Protein Secreting Main Terminal Branch (MTB) Family	pfam db
PF05134	3.A.15	General secretion pathway protein L (GspL)	The Outer Membrane Protein Secreting Main Terminal Branch (MTB) Family	pfam db
PF05157	3.A.15	GSPII_E N-terminal domain	The Outer Membrane Protein Secreting Main Terminal Branch (MTB) Family	pfam db
PF00004	3.A.16	ATPase family associated with various cellular activities (AAA)	The Endoplasmic Reticular Retrotranslocon (ER-RT) Family	auto
PF00213	3.A.2	ATP synthase delta (OSCP) subunit	The H+- or Na+-translocating F-type, V-type and A-type ATPase (F-ATPase) Superfamily	pfam db
PF01496	3.A.2	V-type ATPase 116kDa subunit family	The H+- or Na+-translocating F-type, V-type and A-type ATPase (F-ATPase) Superfamily	pfam db
PF00122	3.A.3	E1-E2 ATPase	The P-type ATPase (P-ATPase) Superfamily	auto
PF00689	3.A.3	Cation transporting ATPase, C-terminus	The P-type ATPase (P-ATPase) Superfamily	pfam db
PF00690	3.A.3	Cation transporter/ATPase, N-terminus	The P-type ATPase (P-ATPase) Superfamily	pfam db
PF00702	3.A.3	haloacid dehalogenase-like hydrolase	The P-type ATPase (P-ATPase) Superfamily	auto
PF02669	3.A.3	K+-transporting ATPase, c chain	The P-type ATPase (P-ATPase) Superfamily	pfam db
PF03814	3.A.3	Potassium-transporting ATPase A subunit	The P-type ATPase (P-ATPase) Superfamily	pfam db
PF02040	3.A.4	Arsenical pump membrane protein	The Arsenite-Antimonite (ArsAB) Efflux Family	pfam db
PF02374	3.A.4	Anion-transporting ATPase	The Arsenite-Antimonite (ArsAB) Efflux Family	pfam db
PF00344	3.A.5	eubacterial secY protein	The General Secretory Pathway (Sec) Family	pfam db
PF00448	3.A.5	SRP54-type protein, GTPase domain	The General Secretory Pathway (Sec) Family	pfam db
PF00584	3.A.5	SecE/Sec61-gamma subunits of protein translocation complex	The General Secretory Pathway (Sec) Family	pfam db
PF02355	3.A.5	Protein export membrane protein	The General Secretory Pathway (Sec) Family	pfam db
PF02699	3.A.5	Preprotein translocase subunit	The General Secretory Pathway (Sec) Family	pfam db
PF02881	3.A.5	SRP54-type protein, helical bundle domain	The General Secretory Pathway (Sec) Family	pfam db
PF02889	3.A.5	Sec63 Brl domain	The General Secretory Pathway (Sec) Family	pfam db
PF02978	3.A.5	Signal peptide binding domain	The General Secretory Pathway (Sec) Family	pfam db
PF03839	3.A.5	Translocation protein Sec62	The General Secretory Pathway (Sec) Family	pfam db
PF03840	3.A.5	Preprotein translocase SecG subunit	The General Secretory Pathway (Sec) Family	pfam db
PF03911	3.A.5	Sec61beta family	The General Secretory Pathway (Sec) Family	pfam db
PF07516	3.A.5	SecA Wing and Scaffold domain	The General Secretory Pathway (Sec) Family	pfam db

PF07517	3.A.5	SecA DEAD-like domain	The General Secretory Pathway (Sec) Family	pfam db
PF00263	3.A.6	Bacterial type II and III secretion system protein	The Type III (Virulence-related) Secretory Pathway (IIISP) Family	pfam db
PF00771	3.A.6	FHIPEP family	The Type III (Virulence-related) Secretory Pathway (IIISP) Family	pfam db
PF00813	3.A.6	FliP family	The Type III (Virulence-related) Secretory Pathway (IIISP) Family	pfam db
PF01311	3.A.6	Bacterial export proteins, family 1	The Type III (Virulence-related) Secretory Pathway (IIISP) Family	pfam db
PF01312	3.A.6	FlhB HrpN YscU SpaS Family	The Type III (Virulence-related) Secretory Pathway (IIISP) Family	pfam db
PF01313	3.A.6	Bacterial export proteins, family 3	The Type III (Virulence-related) Secretory Pathway (IIISP) Family	pfam db
PF01514	3.A.6	Secretory protein of YscJ/FliF family	The Type III (Virulence-related) Secretory Pathway (IIISP) Family	pfam db
PF02108	3.A.6	Flagellar assembly protein FliH	The Type III (Virulence-related) Secretory Pathway (IIISP) Family	pfam db
PF03958	3.A.6	Bacterial type II/III secretion system short domain	The Type III (Virulence-related) Secretory Pathway (IIISP) Family	pfam db
PF04347	3.A.6	Flagellar biosynthesis protein, FliO	The Type III (Virulence-related) Secretory Pathway (IIISP) Family	pfam db
PF00436	3.A.7	Single-strand binding protein family	The Type IV (Conjugal DNA-Protein Transfer or VirB) Secretory Pathway (IVSP) Family	pfam db
PF00437	3.A.7	Type II/IV secretion system protein	The Type IV (Conjugal DNA-Protein Transfer or VirB) Secretory Pathway (IVSP) Family	pfam db
PF02534	3.A.7	TraG/TraD family	The Type IV (Conjugal DNA-Protein Transfer or VirB) Secretory Pathway (IVSP) Family	pfam db
PF03135	3.A.7	CagE, TrbE, VirB family, component of type IV transporter system	The Type IV (Conjugal DNA-Protein Transfer or VirB) Secretory Pathway (IVSP) Family	pfam db
PF03524	3.A.7	Conjugal transfer protein	The Type IV (Conjugal DNA-Protein Transfer or VirB) Secretory Pathway (IVSP) Family	pfam db
PF03743	3.A.7	Bacterial conjugation TrbI-like protein	The Type IV (Conjugal DNA-Protein Transfer or VirB) Secretory Pathway (IVSP) Family	pfam db
PF04335	3.A.7	VirB8 protein	The Type IV (Conjugal DNA-Protein Transfer or VirB) Secretory Pathway (IVSP) Family	pfam db
PF04585	3.A.7	Conjugal transfer protein	The Type IV (Conjugal DNA-Protein Transfer or VirB) Secretory Pathway (IVSP) Family	pfam db
PF04610	3.A.7	TrbL/VirB6 plasmid conjugal transfer protein	The Type IV (Conjugal DNA-Protein Transfer or VirB) Secretory Pathway (IVSP) Family	pfam db
PF04956	3.A.7	TrbC/VIRB2 family	The Type IV (Conjugal DNA-Protein Transfer or VirB) Secretory Pathway (IVSP) Family	pfam db
PF05101	3.A.7	Type IV secretory pathway, VirB3-like protein	The Type IV (Conjugal DNA-Protein Transfer or VirB) Secretory Pathway (IVSP) Family	pfam db

PF05245	3.A.7	Conjugal transfer protein TrbD	The Type IV (Conjugal DNA-Protein Transfer or VirB) Secretory Pathway (IVSP) Family	pfam db
PF01459	3.A.8	Eukaryotic porin	The Mitochondrial Protein Translocase (MPT) Family	pfam db
PF02064	3.A.8	MAS20 protein import receptor	The Mitochondrial Protein Translocase (MPT) Family	pfam db
PF02466	3.A.8	Tim17/Tim22/Tim23 family	The Mitochondrial Protein Translocase (MPT) Family	pfam db
PF04280	3.A.8	Tim44-like domain	The Mitochondrial Protein Translocase (MPT) Family	pfam db
PF04281	3.A.8	Mitochondrial import receptor subunit Tom22	The Mitochondrial Protein Translocase (MPT) Family	pfam db
PF00012	3.A.9	Hsp70 protein	The Chloroplast Envelope Protein Translocase (CEPT or Tic-Toc) Family	pfam db
PF01103	3.A.9	Surface antigen	The Chloroplast Envelope Protein Translocase (CEPT or Tic-Toc) Family	pfam db
PF04278	3.A.9	Tic22-like family	The Chloroplast Envelope Protein Translocase (CEPT or Tic-Toc) Family	pfam db
PF00364	3.B.1	Biotin-requiring enzyme	The Na+-transporting Carboxylic Acid Decarboxylase (NaT-DC) Family	pfam db
PF01039	3.B.1	Carboxyl transferase domain	The Na+-transporting Carboxylic Acid Decarboxylase (NaT-DC) Family	pfam db
PF03977	3.B.1	Na+-transporting methylmalonyl-CoA/oxaloacetate decarboxylase, beta subunit	The Na+-transporting Carboxylic Acid Decarboxylase (NaT-DC) Family	pfam db
PF04277	3.B.1	Oxaloacetate decarboxylase, gamma chain	The Na+-transporting Carboxylic Acid Decarboxylase (NaT-DC) Family	pfam db
PF02007	3.C.1	Tetrahydromethanopterin S-methyltransferase MtrH subunit	The Na+ Transporting Methyltetrahydromethanopterin:Coenzyme M Methyltransferase (NaT-MMM) Family	pfam db
PF04206	3.C.1	Tetrahydromethanopterin S-methyltransferase, subunit E	The Na+ Transporting Methyltetrahydromethanopterin:Coenzyme M Methyltransferase (NaT-MMM) Family	pfam db
PF04207	3.C.1	Tetrahydromethanopterin S-methyltransferase, subunit D	The Na+ Transporting Methyltetrahydromethanopterin:Coenzyme M Methyltransferase (NaT-MMM) Family	pfam db
PF04208	3.C.1	Tetrahydromethanopterin S-methyltransferase, subunit A	The Na+ Transporting Methyltetrahydromethanopterin:Coenzyme M Methyltransferase (NaT-MMM) Family	pfam db
PF04210	3.C.1	Tetrahydromethanopterin S-methyltransferase, subunit G	The Na+ Transporting Methyltetrahydromethanopterin:Coenzyme M Methyltransferase (NaT-MMM) Family	pfam db
PF04211	3.C.1	Tetrahydromethanopterin S-methyltransferase, subunit C	The Na+ Transporting Methyltetrahydromethanopterin:Coenzyme M Methyltransferase (NaT-MMM) Family	pfam db
PF05440	3.C.1	Tetrahydromethanopterin S-methyltransferase subunit B	The Na+ Transporting Methyltetrahydromethanopterin:Coenzyme M Methyltransferase (NaT-MMM) Family	pfam db
PF00146	3.D.1	NADH dehydrogenase	The Proton-translocating NADH Dehydrogenase (NDH) Family	pfam db
PF00329	3.D.1	Respiratory-chain NADH dehydrogenase, 30 Kd subunit	The Proton-translocating NADH Dehydrogenase (NDH) Family	pfam db
PF00346	3.D.1	Respiratory-chain NADH dehydrogenase, 49 Kd subunit	The Proton-translocating NADH Dehydrogenase (NDH) Family	pfam db

PF00361	3.D.1	NADH-Ubiquinone/plastoquinone (complex I), various chains	The Proton-translocating NADH Dehydrogenase (NDH) Family	pfam db
PF00374	3.D.1	Nickel-dependent hydrogenase	The Proton-translocating NADH Dehydrogenase (NDH) Family	pfam db
PF00420	3.D.1	NADH-ubiquinone/plastoquinone oxidoreductase chain 4L	The Proton-translocating NADH Dehydrogenase (NDH) Family	pfam db
PF00499	3.D.1	NADH-ubiquinone/plastoquinone oxidoreductase chain 6	The Proton-translocating NADH Dehydrogenase (NDH) Family	pfam db
PF00507	3.D.1	NADH-ubiquinone/plastoquinone oxidoreductase, chain 3	The Proton-translocating NADH Dehydrogenase (NDH) Family	pfam db
PF00662	3.D.1	NADH-Ubiquinone oxidoreductase (complex I), chain 5 N-terminus	The Proton-translocating NADH Dehydrogenase (NDH) Family	pfam db
PF01058	3.D.1	NADH ubiquinone oxidoreductase, 20 Kd subunit	The Proton-translocating NADH Dehydrogenase (NDH) Family	pfam db
PF01257	3.D.1	Respiratory-chain NADH dehydrogenase 24 Kd subunit	The Proton-translocating NADH Dehydrogenase (NDH) Family	pfam db
PF01512	3.D.1	Respiratory-chain NADH dehydrogenase 51 Kd subunit	The Proton-translocating NADH Dehydrogenase (NDH) Family	pfam db
PF01899	3.D.1	Na+/H+ ion antiporter subunit	The Proton-translocating NADH Dehydrogenase (NDH) Family	pfam db
PF03334	3.D.1	Na+/H+ antiporter subunit	The Proton-translocating NADH Dehydrogenase (NDH) Family	pfam db
PF04039	3.D.1	Domain related to MnhB subunit of Na+/H+ antiporter	The Proton-translocating NADH Dehydrogenase (NDH) Family	pfam db
PF04066	3.D.1	Multiple resistance and pH regulation protein F (MrpF / PhaF)	The Proton-translocating NADH Dehydrogenase (NDH) Family	pfam db
PF01262	3.D.2	Alanine dehydrogenase/PNT, C-terminal domain	The Proton-translocating Transhydrogenase (PTH) Family	pfam db
PF02233	3.D.2	NAD(P) transhydrogenase beta subunit	The Proton-translocating Transhydrogenase (PTH) Family	auto
PF05222	3.D.2	Alanine dehydrogenase/PNT, N-terminal domain	The Proton-translocating Transhydrogenase (PTH) Family	pfam db
PF00032	3.D.3	Cytochrome b(C-terminal)/b6/petD	The Proton-translocating Quinol:Cytochrome c Reductase (QCR) Superfamily	pfam db
PF00355	3.D.3	Rieske [2Fe-2S] domain	The Proton-translocating Quinol:Cytochrome c Reductase (QCR) Superfamily	pfam db
PF01333	3.D.3	Apocytochrome F, C-terminal	The Proton-translocating Quinol:Cytochrome c Reductase (QCR) Superfamily	pfam db
PF02167	3.D.3	Cytochrome C1 family	The Proton-translocating Quinol:Cytochrome c Reductase (QCR) Superfamily	pfam db
PF02921	3.D.3	Ubiquinol cytochrome reductase transmembrane region	The Proton-translocating Quinol:Cytochrome c Reductase (QCR) Superfamily	pfam db
PF00115	3.D.4	Cytochrome C and Quinol oxidase polypeptide I	The Proton-translocating Cytochrome Oxidase (COX) Superfamily	pfam db
PF00116	3.D.4	Cytochrome C oxidase subunit II, periplasmic domain	The Proton-translocating Cytochrome Oxidase (COX) Superfamily	pfam db
PF00510	3.D.4	Cytochrome c oxidase subunit III	The Proton-translocating Cytochrome Oxidase (COX) Superfamily	pfam db
PF01040	3.D.4	UbiA prenyltransferase family	The Proton-translocating Cytochrome Oxidase (COX) Superfamily	pfam db

PF02790	3.D.4	Cytochrome C oxidase subunit II, transmembrane domain	The Proton-translocating Cytochrome Oxidase (COX) Superfamily	pfam db
PF03626	3.D.4	Prokaryotic Cytochrome C oxidase subunit IV	The Proton-translocating Cytochrome Oxidase (COX) Superfamily	pfam db
PF00175	3.D.5	Oxidoreductase NAD-binding domain	The Na+-translocating NADH:Quinone Dehydrogenase (Na-NDH) Family	pfam db
PF03116	3.D.5	NQR2, RnfD, RnfE family	The Na+-translocating NADH:Quinone Dehydrogenase (Na-NDH) Family	pfam db
PF05896	3.D.5	Na(+)-translocating NADH-quinone reductase subunit A (NQRA)	The Na+-translocating NADH:Quinone Dehydrogenase (Na-NDH) Family	pfam db
PF01512	3.D.6	Respiratory-chain NADH dehydrogenase 51 Kd subunit	The Putative Ion (H+ or Na+)-translocating NADH:Ferredoxin Oxidoreductase (NFO) Family	pfam db
PF03116	3.D.6	NQR2, RnfD, RnfE family	The Putative Ion (H+ or Na+)-translocating NADH:Ferredoxin Oxidoreductase (NFO) Family	pfam db
PF03658	3.D.6	Uncharacterised protein family (UPF0125)	The Putative Ion (H+ or Na+)-translocating NADH:Ferredoxin Oxidoreductase (NFO) Family	pfam db
PF00374	3.D.7	Nickel-dependent hydrogenase	The H2:Heterodisulfide Oxidoreductase (HHO) Family	pfam db
PF01058	3.D.7	NADH ubiquinone oxidoreductase, 20 Kd subunit	The H2:Heterodisulfide Oxidoreductase (HHO) Family	pfam db
PF01979	3.D.8	Amidohydrolase family	The Na+- or H+-Pumping Formyl Methanofuran Dehydrogenase (FMF-DH) Family	manual
PF02663	3.D.8	Tungsten formylmethanofuran dehydrogenase, subunit E, FwdE	The Na+- or H+-Pumping Formyl Methanofuran Dehydrogenase (FMF-DH) Family	pfam db
PF00146	3.D.9	NADH dehydrogenase	The H+-translocating F420H2 Dehydrogenase (F420H2DH) Family	pfam db
PF00329	3.D.9	Respiratory-chain NADH dehydrogenase, 30 Kd subunit	The H+-translocating F420H2 Dehydrogenase (F420H2DH) Family	pfam db
PF00361	3.D.9	NADH-Ubiquinone/plastoquinone (complex I), various chains	The H+-translocating F420H2 Dehydrogenase (F420H2DH) Family	pfam db
PF00420	3.D.9	NADH-ubiquinone/plastoquinone oxidoreductase chain 4L	The H+-translocating F420H2 Dehydrogenase (F420H2DH) Family	pfam db
PF00499	3.D.9	NADH-ubiquinone/plastoquinone oxidoreductase chain 6	The H+-translocating F420H2 Dehydrogenase (F420H2DH) Family	pfam db
PF00507	3.D.9	NADH-ubiquinone/plastoquinone oxidoreductase, chain 3	The H+-translocating F420H2 Dehydrogenase (F420H2DH) Family	pfam db
PF00662	3.D.9	NADH-Ubiquinone oxidoreductase (complex I), chain 5 N-terminus	The H+-translocating F420H2 Dehydrogenase (F420H2DH) Family	pfam db
PF01058	3.D.9	NADH ubiquinone oxidoreductase, 20 Kd subunit	The H+-translocating F420H2 Dehydrogenase (F420H2DH) Family	pfam db
PF04422	3.D.9	Coenzyme F420 hydrogenase/dehydrogenase, beta subunit N terminus	The H+-translocating F420H2 Dehydrogenase (F420H2DH) Family	pfam db
PF04432	3.D.9	Coenzyme F420 hydrogenase/dehydrogenase, beta subunit C terminus	The H+-translocating F420H2 Dehydrogenase (F420H2DH) Family	pfam db
PF01036	3.E.1	Bacteriorhodopsin	The Ion-translocating Microbial Rhodopsin (MR) Family	pfam db
PF00124	3.E.2	Photosynthetic reaction centre protein	The Photosynthetic Reaction Center (PRC) Family	pfam db

PF00355	3.E.2	Rieske [2Fe-2S] domain	The Photosynthetic Reaction Center (PRC) Family	pfam db
PF00421	3.E.2	Photosystem II protein	The Photosynthetic Reaction Center (PRC) Family	pfam db
PF03967	3.E.2	Photosynthetic reaction centre, H-chain N-terminal region	The Photosynthetic Reaction Center (PRC) Family	pfam db
PF05239	3.E.2	PRC-barrel domain	The Photosynthetic Reaction Center (PRC) Family	pfam db
PF00358	4.A.1	phosphoenolpyruvate-dependent sugar phosphotransferase system, EIIA 1	The PTS Glucose-Glucoside (Glc) Family	pfam db
PF00367	4.A.1	phosphotransferase system, EIIB	The PTS Glucose-Glucoside (Glc) Family	pfam db
PF02378	4.A.1	Phosphotransferase system, EIIC	The PTS Glucose-Glucoside (Glc) Family	pfam db
PF00359	4.A.2	Phosphoenolpyruvate-dependent sugar phosphotransferase system, EIIA 2	The PTS Fructose-Mannitol (Fru) Family	pfam db
PF02302	4.A.2	PTS system, Lactose/Cellobiose specific IIB subunit	The PTS Fructose-Mannitol (Fru) Family	pfam db
PF02378	4.A.2	Phosphotransferase system, EIIC	The PTS Fructose-Mannitol (Fru) Family	pfam db
PF02379	4.A.2	PTS system, Fructose specific IIB subunit	The PTS Fructose-Mannitol (Fru) Family	pfam db
PF02255	4.A.3	PTS system, Lactose/Cellobiose specific IIA subunit	The PTS Lactose-N,N'-Diacetylchitobiose-beta-glucoside (Lac) Family	pfam db
PF02302	4.A.3	PTS system, Lactose/Cellobiose specific IIB subunit	The PTS Lactose-N,N'-Diacetylchitobiose-beta-glucoside (Lac) Family	pfam db
PF02378	4.A.3	Phosphotransferase system, EIIC	The PTS Lactose-N,N'-Diacetylchitobiose-beta-glucoside (Lac) Family	pfam db
PF03829	4.A.4	PTS system glucitol/sorbitol-specific IIA component	The PTS Glucitol (Gut) Family	pfam db
PF00359	4.A.5	Phosphoenolpyruvate-dependent sugar phosphotransferase system, EIIA 2	The PTS Galactitol (Gat) Family	pfam db
PF02302	4.A.5	PTS system, Lactose/Cellobiose specific IIB subunit	The PTS Galactitol (Gat) Family	pfam db
PF03830	4.A.6	PTS system sorbose subfamily IIB component	The PTS Mannose-Fructose-Sorbose (Man) Family	pfam db
PF02302	4.A.7	PTS system, Lactose/Cellobiose specific IIB subunit	The PTS L-Ascorbate (L-Asc) Family	pfam db
PF04215	4.A.7	Putative sugar-specific permease, SgaT/UlaA	The PTS L-Ascorbate (L-Asc) Family	pfam db
PF00501	4.C.1	AMP-binding enzyme	The Proposed Fatty Acid Transporter (FAT) Family	auto
PF02683	5.A.1	Cytochrome C biogenesis protein transmembrane region	The Disulfide Bond Oxidoreductase D (DsbD) Family	pfam db
PF02600	5.A.2	Disulfide bond formation protein DsbB	The Disulfide Bond Oxidoreductase B (DsbB) Family	pfam db
PF00384	5.A.3	Molybdopterin oxidoreductase	The Prokaryotic Molybdopterin-containing Oxidoreductase (PMO) Family	auto
PF02665	5.A.3	Nitrate reductase gamma subunit	The Prokaryotic Molybdopterin-containing Oxidoreductase (PMO) Family	pfam db
PF03188	5.A.4	Eukaryotic cytochrome b561		pfam db
PF08022	5.B.1	FAD-binding domain	The Phagocyte (gp91phox) NADPH Oxidase Family	auto
PF08030	5.B.1	Ferric reductase NAD binding domain	The Phagocyte (gp91phox) NADPH Oxidase Family	auto
PF03188	5.B.2	Eukaryotic cytochrome b561	The Eukaryotic Cytochrome b561 (Cytb561) Family	manual
PF00529	8.A.1	HlyD family secretion protein	The Membrane Fusion Protein (MFP)	auto

			Family	
PF02060	8.A.10	Slow voltage-gated potassium channel	The Slow Voltage-gated K+ Channel Accessory Protein (MinK) Family	pfam db
PF04272	8.A.11	Phospholamban	The Immunophilin-like Prolyl:peptidyl Isomerase Regulator (I-PPI) Family	pfam db
PF03185	8.A.14	Calcium-activated potassium channel, beta subunit	The Ca2+-activated K+ Channel Auxiliary Subunit Slowpoke-beta (Slobeta) Family	pfam db
PF00822	8.A.16	PMP-22/EMP/MP20/Claudin family	The Ca+ Channel Auxiliary Subunit gamma1-gamma8 (CCAgamma) Family	pfam db
PF01145	8.A.21	SPFH domain / Band 7 family	The Stomatin/Podocin/Band 7/Nephrosis.2/SPFH (Stomatin) Family	manual
PF00373	8.A.25	FERM central domain	The Ezrin/Radixin/Moesin (Ezrin) Family	manual
PF00769	8.A.25	Ezrin/radixin/moesin family	The Ezrin/Radixin/Moesin (Ezrin) Family	manual
PF02706	8.A.3	Chain length determinant protein	The Cytoplasmic Membrane-Periplasmic Auxiliary-1 (MPA1) Protein with Cytoplasmic (C) Domain (MPA1-C or MPA1+C) Family	auto
PF00248	8.A.5	Aldo/keto reductase family	The Voltage-gated K+ Channel beta-subunit (Kvbeta) Family	pfam db
PF00391	8.A.7	PEP-utilising enzyme, mobile domain	The Phosphotransferase System Enzyme I (EI) Family	manual
PF02896	8.A.7	PEP-utilising enzyme, TIM barrel domain	The Phosphotransferase System Enzyme I (EI) Family	manual
PF05524	8.A.7	PEP-utilising enzyme, N-terminal	The Phosphotransferase System Enzyme I (EI) Family	manual
PF00537	8.B.1	Scorpion toxin-like domain	The Long (4C-C) Scorpion Toxin (L-ST) Superfamily	auto
PF07740	8.B.5	Spider potassium channel inhibitory toxin	The Na+/K+/Ca2+ Channel Targeting Tarantula Huwentoxin (THT) Family	auto
PF03381	9.A.1	LEM3 (ligand-effect modulator 3) family / CDC50 family	The Non ABC Multidrug Exporter (N-MDE) Family	manual
PF03239	9.A.10	Iron permease FTR1 family	The Iron/Lead Transporter (ILT) Superfamily	pfam db
PF03323	9.A.11	Bacillus/Clostridium GerA spore germination protein	The Dipicolinic Acid Transporter (DPA-T) Family	auto
PF04145	9.A.11	Ctr copper transporter family	The Dipicolinic Acid Transporter (DPA-T) Family	pfam db
PF04145	9.A.12	Ctr copper transporter family	The Peptidoglycolipid Addressing Protein (GAP) Family	pfam db
PF01299	9.A.16	Lysosome-associated membrane glycoprotein (Lamp)	The Lysosomal Protein Import (LPI) Family	pfam db
PF03239	9.A.17	Iron permease FTR1 family	The Integral Membrane Peroxisomal Protein Importer-2 (PPI2) Family	pfam db
PF08610	9.A.17	Peroxisomal membrane protein (Pex16)	The Integral Membrane Peroxisomal Protein Importer-2 (PPI2) Family	auto
PF05992	9.A.18	SbmA/BacA-like family	The Putative Peptide Uptake or Activated Fatty Acid Export Permease (PUP) Family	pfam db
PF00571	9.A.19	CBS domain pair	The Mg2+ Transporter-E (MgtE) Family	auto
PF01769	9.A.19	Divalent cation transporter	The Mg2+ Transporter-E (MgtE) Family	pfam db
PF00403	9.A.2	Heavy-metal-associated domain	The MerTP Mercuric Ion (Hg2+) Permease (MerTP) Family	pfam db
PF06963	9.A.23	Ferroportin1 (FPN1)	The Ferroportin (FP) Family	manual
PF04346	9.A.28	Ethanolamine utilisation protein, EutH	The Ethanolamine Facilitator (EAF) Family	manual
PF03203	9.A.3	MerC mercury resistance protein	The MerC Mercuric Ion (Hg2+) Uptake (MerC) Family	pfam db

PF07724	9.A.34	ATPase family associated with various cellular activities (AAA)	The Putative Type VI Symbiosis/Virulence Secretory Pathway (VISP) Family	manual
PF05653	9.A.35	Protein of unknown function (DUF803)	The NIPA Mg2+ Uptake Permease (NIPA) Family	auto
PF04973	9.A.4	Nicotinamide mononucleotide transporter	The YggT or Fanciful K+ Uptake-B (FkuB; YggT) Family	pfam db
PF01580	9.A.44	FtsK/SpoIIIE family	The EsxA/EsxB Protein Secretion Pathway (EPSP) Family	auto
PF00168	9.A.48	C2 domain	The Unconventional Protein Secretion (UPS) System	auto
PF00191	9.A.48	Annexin	The Unconventional Protein Secretion (UPS) System	auto
PF00004	9.A.5	ATPase family associated with various cellular activities (AAA)	The Peroxisomal Protein Importer (PPI) Family	auto
PF04695	9.A.5	Peroxisomal membrane anchor protein (Pex14p) conserved region	The Peroxisomal Protein Importer (PPI) Family	pfam db
PF04757	9.A.5	Pex2 / Pex12 amino terminal region	The Peroxisomal Protein Importer (PPI) Family	pfam db
PF01926	9.A.8	GTPase of unknown function	The Ferrous Iron Uptake (FeoB) Family	auto
PF02421	9.A.8	Ferrous iron transport protein B	The Ferrous Iron Uptake (FeoB) Family	pfam db
PF07664	9.A.8	Ferrous iron transport protein B C terminus	The Ferrous Iron Uptake (FeoB) Family	pfam db
PF07670	9.A.8	Nucleoside recognition	The Ferrous Iron Uptake (FeoB) Family	auto
PF04120	9.A.9	Low affinity iron permease	The Low Affinity Fe2+ Transporter (FeT) Family	pfam db
PF01914	9.B.10	MarC family integral membrane protein	The Putative Multiple antibiotic resistance MarC Family	pfam db
PF08085	9.B.13	Entericidin EcnA/B family	The Putative Pore-forming Entericidin (ECN) Family	manual
PF01578	9.B.14	Cytochrome C assembly protein	The Putative Heme Handling Protein (HHP) Family	pfam db
PF00501	9.B.17	AMP-binding enzyme	The VAMP-associated protein (VAP) Family	manual
PF02699	9.B.18	Preprotein translocase subunit	The SecDF-associated Single Transmembrane Protein, YajC (YajC) Family	pfam db
PF02308	9.B.20	MgtC family	The Putative Mg2+ Transporter-C (MgtC) Family	pfam db
PF01491	9.B.21	Frataxin-like domain	The Frataxin (Frataxin) Family	manual
PF01594	9.B.22	Domain of unknown function DUF20	The Putative Thiamin Transporter (PTT) Family	pfam db
PF01027	9.B.24	Uncharacterised protein family UPF0005	The Testis-Enhanced Gene Transfer (TEGT) Family	pfam db
PF03649	9.B.25	Uncharacterised protein family (UPF0014)	The YbbM (YbbM) Family	pfam db
PF09335	9.B.27	SNARE associated Golgi protein	The YdjX-Z (YdjX-Z) Family	manual
PF01098	9.B.3	Cell cycle protein	The Putative Bacterial Murein Precursor Exporter (MPE) Family	pfam db
PF03006	9.B.30	Haemolysin-III related	The Hly III (Hly III) Family	pfam db
PF02660	9.B.31	Domain of unknown function DUF	The YqiH (YqiH) Family	pfam db
PF00535	9.B.32	Glycosyl transferase family 2	The Putative Vectorial Glycosyl Polymerization (VGP) Family	pfam db
PF03552	9.B.32	Cellulose synthase	The Putative Vectorial Glycosyl Polymerization (VGP) Family	manual
PF01184	9.B.33	GPR1/FUN34/yaaH family	The YaaH (YaaH) Family	pfam db

PF00576	9.B.35	HIUase/Transthyretin family	The Putative Thyronine-Transporting Transthyretin (Transthyretin) Family	pfam db
PF03471	9.B.37	Transporter associated domain		pfam db
PF01130	9.B.39	CD36 family	The Long Chain Fatty Acid Translocase (lcFAT) Family	manual
PF01027	9.B.4	Uncharacterised protein family UPF0005	The Universal Stress Protein-B (UspB) Family	pfam db
PF05976	9.B.4	Bacterial membrane protein of unknown function (DUF893)	The Universal Stress Protein-B (UspB) Family	pfam db
PF02402	9.B.41	Lysis protein	The Colicin Lysis Protein (CLP) Family	pfam db
PF05360	9.B.44	yiaA/B two helix domain	The YiaA-YiaB (YiaAB) Family	pfam db
PF02694	9.B.45	Uncharacterised BCR, YnfA/UPF0060 family	The YnfA (YnfA) Family	pfam db
PF04647	9.B.46	Accessory gene regulator B	The Staphylococcus aureus Putative Quorum Sensing Peptide Exporter, AgrB (AgrB) Family	pfam db
PF03783	9.B.47	Curli production assembly/transport component CsgG		pfam db
PF02447	9.B.49	GntP family permease	The Unknown IT-2 (UIT2) Family	pfam db
PF03606	9.B.50	C4-dicarboxylate anaerobic carrier	The Unknown IT-3 (UIT3) Family	pfam db
PF02554	9.B.59	Carbon starvation protein CstA	The Putative Peptide Transporter Carbon Starvation CstA (CstA) Family	pfam db
PF01848	9.B.6	Hok/gef family	The Toxic Hok/Gef Protein (Hok/Gef) Family	pfam db
PF05425	9.B.62	Copper resistance protein D	The Copper Resistance (CopD) Family	manual
PF01852	9.B.64	START domain	The Putative Cholesterol Transporter (Start1) Family	manual
PF04932	9.B.67	O-Antigen Polymerase	The Putative Inorganic Carbon (HCO3-) Transporter/O-antigen Polymerase (ICT/OAP) Family	auto
PF03209	9.B.7	PUCC protein	The Putative Sulfate Transporter (CysZ) Family	pfam db
PF03176	9.B.74	MMPL family	The Phage Infection Protein (PIP) Family	auto
PF03248	9.B.82	Rer1 family	Endoplasmic Reticulum Retrieval Protein1 (Putative Heavy Metal Transporter) (Rer1) Family	auto
