# Supplementary material for: TransportTP: A two-phase classification approach for membrane transporter prediction and characterization
Source: BMC Bioinformatics. 2009 Dec 14;10:418. doi: 10.1186/1471-2105-10-418 (PMC3087344; doi:10.1186/1471-2105-10-418)
Supplement: Additional file 8 — The keywords adopted in the text-mining program to detect non-transporter functions in Swiss-Prot database. This PDF displays the keywords adopted in the text-mining program to detect non-transporter functions hit by unknown proteins in Swiss-prot Database. [file 1471-2105-10-418-S8.PDF]

INHIBITOR,
TRANSCRIPTION FACTOR,
NUCLEASE,
CYTOPLASMIC,
BIOSYNTHESIS,
RIBOSOMAL,
RIBONUCLEASE,
TRANSLATION,
POLYMERASE,
SPLICING,
HISTONE,
BIOGENESIS,
REPAIR,
RECOMBINATION,
KINASE,
ZINC FINGER,
TRANSPOSON,
CYSTEINE-RICH,
LEUCINE-RICH,
ARGONAUTE,
COPIA,
VICILIN,
REVERSE TRANSCRIPTASE,
MITOGEN-ACTIVATED,
CHROMATIN,
HOMEOBOX,
CYCLIN-DEPENDENT,
SYSTEMIN,
LIGASE,
UBIQUIN,
F-BOX,
SUBTILISIN,
EXPANSIN,
RHO,
DNA REPAIR,
CYTOCHROME,
RETRANSPOSABLE ELEMENT,
TWO-COMPONENT,
MALE STERILITY
